# How does the SARS-CoV-2 reinfection rate change over time? The global evidence from systematic review and meta-analysis

**DOI:** 10.1186/s12879-024-09225-z

**Published:** 2024-03-21

**Authors:** Ying Chen, Wenhui Zhu, Xinyue Han, Miaoshuang Chen, Xin Li, Haiping Huang, Mengyuan Zhang, Rongjie Wei, Huadong Zhang, Changhong Yang, Tao Zhang

**Affiliations:** 1 Department of Epidemiology and Health Statistics, West China School of Public Health and West China Fourth Hospital, Sichuan University, Chengdu, Sichuan Province China; 2 Sichuan Center for Disease Control and Prevention, Chengdu, Sichuan Province China; 3 Chongqing Center for Disease Control and Prevention, Chongqing, China

**Keywords:** Time-varying reinfection rate, Meta-analysis, Meta-regression, SARS-CoV-2, COVID-19

## Abstract

**Background:**

There is a significant increase in the number of SARS-CoV-2 reinfection reports in various countries. However, the trend of reinfection rate over time is not clear.

**Methods:**

We searched PubMed, Web of Science, Medline, Embase, Cochrane Central Register of Controlled Trials, China National Knowledge Infrastructure, and Wanfang for cohort studies, case-control studies, and cross-sectional studies up to March 16, 2023, to conduct a meta-analysis of global SARS-CoV-2 reinfection rate. Subgroup analyses were performed for age, country, study type, and study population, and time-varying reinfection rates of SARS-CoV-2 were estimated using meta-regression. The risk of bias was assessed using the Newcastle-Ottawa Scale and the Joanna Briggs Institute critical appraisal tool.

**Result:**

A total of 55 studies involving 111,846 cases of SARS-CoV-2 reinfection were included. The pooled SARS-CoV-2 reinfection rate was 0.94% (95% *CI*: 0.65 -1.35%). In the subgroup analyses, there were statistically significant differences in the pooled reinfection rates by reinfection variant, and study type (*P* < 0.05). Based on meta-regression, the reinfection rate fluctuated with time.

**Conclusion:**

Meta-regression analysis found that the overall reinfection rate increased and then decreased over time, followed by a period of plateauing and then a trend of increasing and then decreasing, but the peak of the second wave of reinfection rate was lower than the first wave. SARS-CoV-2 is at risk of reinfection and the Omicron variant has a higher reinfection rate than other currently known variants. The results of this study could help guide public health measures and vaccination strategies in response to the Coronavirus Disease 2019 (COVID-19) pandemic.

**Supplementary Information:**

The online version contains supplementary material available at 10.1186/s12879-024-09225-z.

## Introduction

Since the onset of Coronavirus Disease 2019 (COVID-19), SARS-CoV-2 infection has been circulating globally, with approximately six peak waves of outbreaks worldwide before May 26, 2023, with a cumulative number of confirmed SARS-CoV-2 infections of 76,689,575 and a total number of deaths of 6,935,889 [1], posing a serious threat to the health of populations in all countries. On May 5, 2023 [1, 2], the World Health Organization (WHO) declared that the COVID-19 epidemic no longer constituted a public health emergency of international concern. However, it is undeniable that the COVID-19 pandemic caused a large number of deaths and was devastating. The fact that SARS-CoV-2 is still mutating indicates that it remains a global health threat. The WHO study published in Nature [1, 3] also suggested that accurately tracking SARS-CoV-2 and its impact had been challenging.

Reinfections have been reported continuously since the first wave of the COVID-19 epidemic, and especially after the Omicron variant became a major prevalent variant worldwide, there has been a significant increase in the number of reported SARS-CoV-2 reinfections globally [4]. Bastard et al. [5] showed a low rate of SARS-CoV-2 reinfection before the Omicron epidemic (1% of all confirmed COVID-19 cases), but a dramatic increase in reinfection occurred after the emergence and spread of the Omicron variant (from December 2021 to February 2022), accounting for more than 4% of all COVID-19 cases diagnosed in mid-February 2022. Additionally, an Italian study [6] revealed that the risk of reinfection during the Omicron epidemic was 4.89 times higher than during the Delta epidemic (95% *CI*: 4.19–5.72, *P* < 0.001).When comparing the severity of SARS-CoV-2 reinfection cases, the majority of patients experienced milder symptoms during the second infection in comparison to the first one [7]. However, some reinfection cases were more severe than the first, requiring hospitalization or sequelae of infection [8], and even leading to the death of the reinfected patient [8]. Arslan et al. [9]found a higher rate of intensive care unit admissions for reinfection (2.9%) than for the first infection (0.5%). Consequently, SARS-CoV-2 reinfection remains a significant global public health concern.

At present, some studies have summarized the global reinfection rate of SARS-CoV-2 through meta-analysis. However, the previous meta-analysis only performed subgroup analysis on age, country, or disease severity, and the reinfection rate was a fixed value. The time trend of the reinfection rate is not clear [10–12]. Otherwise, the SARS-CoV-2 will continue to change over time, and their transmission risks [13–15] and clinical deterioration of the disease are not consistent, resulting in reinfection will not be maintained at a fixed level. Therefore, the resulting pooled reinfection rates may be highly biased for previous meta-analyses did not take the effect of time into account. Moreover, there is still no clear global definition of SARS-CoV-2 reinfection. Some studies defined reinfection as the time interval between two positive tests needing to be more than 90 days [16, 17], while some studies defined reinfection as the time interval only needing to be more than 30 days [18, 19], and it was difficult to directly compare reinfection rates under different definitions with large differences [20]. Therefore, the aim of this study was to conduct a meta-analysis to describe and summarize the current reinfection rates in different regions and among diverse populations. Furthermore, the study sought to examine trends in reinfection rates over time, so as to provide reference for the development of global unified reinfection standards and reinfection measures in the future.

## Methods

### Study design

A meta-regression analysis was conducted on literature reporting cases of SARS-CoV-2 reinfection globally to estimate the incidence of reinfection of SARS-CoV-2 (as of March 16, 2023), and reported according to the Preferred Reporting Items for Systematic Reviews and Meta-Analyses (PRISMA) guidelines (Additional file 1). This systematic review and meta-analysis was registered with PROSPERO (CRD42023411778).

We searched for papers published on PubMed, Web of Science, Medline(Ovid), Embase(Ovid), Cochrane Central Register of Controlled Trials, China National Knowledge Infrastructure(CNKI), Wanfang using “COVID-19”, “2019-nCoV”, “SARS-COV-2”, “Severe Acute Respiratory Syndrome Coronavirus 2”, “reinfection”, “recurrence”, “repeat positive” and “repeat infections” as keywords, from January 1, 2020 to March 16, 2023 (Additional file 2).

### Selection criteria and data extraction

To avoid language bias, the search was not limited to any language. We conducted preliminary screening based on the English title and English abstract provided by non-English articles. Further, in the screening process, if the included article was neither in English nor in Chinese and did not provide an English abstract or full text, we used an online translator (Google Translate) for translation. There were no non-English articles in the final articles included in this study. The inclusion criteria were: (1) Conformed to research questions, i.e., studies that estimated the reinfection rate of SARS-CoV-2 or we could calculate it based on the data provided in the paper; (2) The study design included cohort study, case-control study, and descriptive study; (3) The study fited our working definition of SARS-CoV-2 reinfection. SARS-CoV-2 reinfection working definition in this study was based on a positive laboratory result at least 90 days after laboratory confirmation of primary infection(laboratory testing methods include reverse transcription–polymerase chain reaction or rapid antigen test, also called Lateral Flow Devices, and so on), advised from WHO and US Centers for Disease Control and Prevention [21, 22]. The definition of reinfection was explained in the “Discussion” section; (4) Original research. The exclusion criteria were: (1) The sample size was less than 20; (2) Belonged to one of the following types of studies: editorial, case report, case series study, systematic review, meta-analysis, animal experiment, news report; (3) Secondary reporting or articles on repeated studies of the same population; (4) Grey literature, including a range of documents not controlled by commercial publishing organizations. The reviewers independently (CY, Z-WH, H-XY, C-MS, LX, H-HP) screened the literature based on inclusion and exclusion criteria. Conflicts were resolved by other independent reviewers (ZT Y-CH).

The complete information extracted included the first author, study period, country, study population, sample size, study design, reinfected variants, time between two positive tests (days), age, reinfection rate, and the number of reinfections. Citations and characteristics for all included studies and all data inputs were shown in Additional file 3. If the paper did not mention the reinfection variant, we classified the reinfection variants based on the WHO standards according to the study period. The discovery times of different variants were shown in Additional file 3.

We used the Newcastle-Ottawa Scale (NOS) to evaluate the literature quality of cohort and case-control studies, and the Joanna Briggs Institute critical appraisal tool (JBI) for cross-sectional studies (Additional file 4). Risk bias assessment was performed by two independent reviewers (H-XY, C-MS), and inconsistencies were judged by other independent reviewers (CY, Z-WH). When the star rating in NOS was less than 3, or the number of “yes” in JBI was less than 5 [23], we categorized the paper as “high risk of bias”.

### Statistical method

We used meta-analysis to evaluate the pooled rate of SARS-CoV-2 reinfection in the included studies. Subgroup analysis was also performed to obtain reinfection rates and forest plots according to age, variant, and country. Meta-analysis is a method to obtain weighted average results from various studies. In addition to pooling effect sizes, meta-analysis can also be used to estimate disease frequencies, such as incidence and prevalence. However, when the rate is close to 0 or 1, two problems will occur in the meta-regression: the confidence interval may exceed the interval of 0–1, and the variance is squeezed towards 0. Therefore, we converted the reinfection rate to an approximate normal distribution. Four transformations were taken: log transformation, logit transformation, arcsine transformation, and double arcsine transformation. Through the hypothesis test after the transformation, we decided to use the logit transformation (see Additional file 5 for specific results).

Heterogeneity among studies was assessed due to differences in variant and location between studies. Heterogeneity between studies was expressed using Cochran’s *Q* statistic test (*P* < 0.05) as well as ${I}^{2}$> 50%. If heterogeneity was low, a fixed-effects model was used to combine rates and correction estimates from different studies. Otherwise, a random-effects model was used. We used the Akaike information criterion to determine the variable form of the meta-regression. Moreover, we used two methods for sensitivity analysis: the first method involved recalculating the combined values by excluding highly biased papers and comparing the differences in results before and after exclusion; the second method involved sequentially excluding each included paper and analyzing whether there were significant differences in results before and after exclusion.

In addition, considering the change in the reinfection rate over time, we constructed a multiple meta-regression using the time interval between two positive tests as the independent variable, to estimate the time-varying reinfection rate. Subgroup analyses were performed by meta-regression to fit stratified rates of reinfection based on variables such as variant and country, and we also used spline regression to fit time-varying reinfection rates that did not account for heterogeneity. All analyses were performed using R statistical software (version 4.2.3).

## Results

**Fig. 1:**
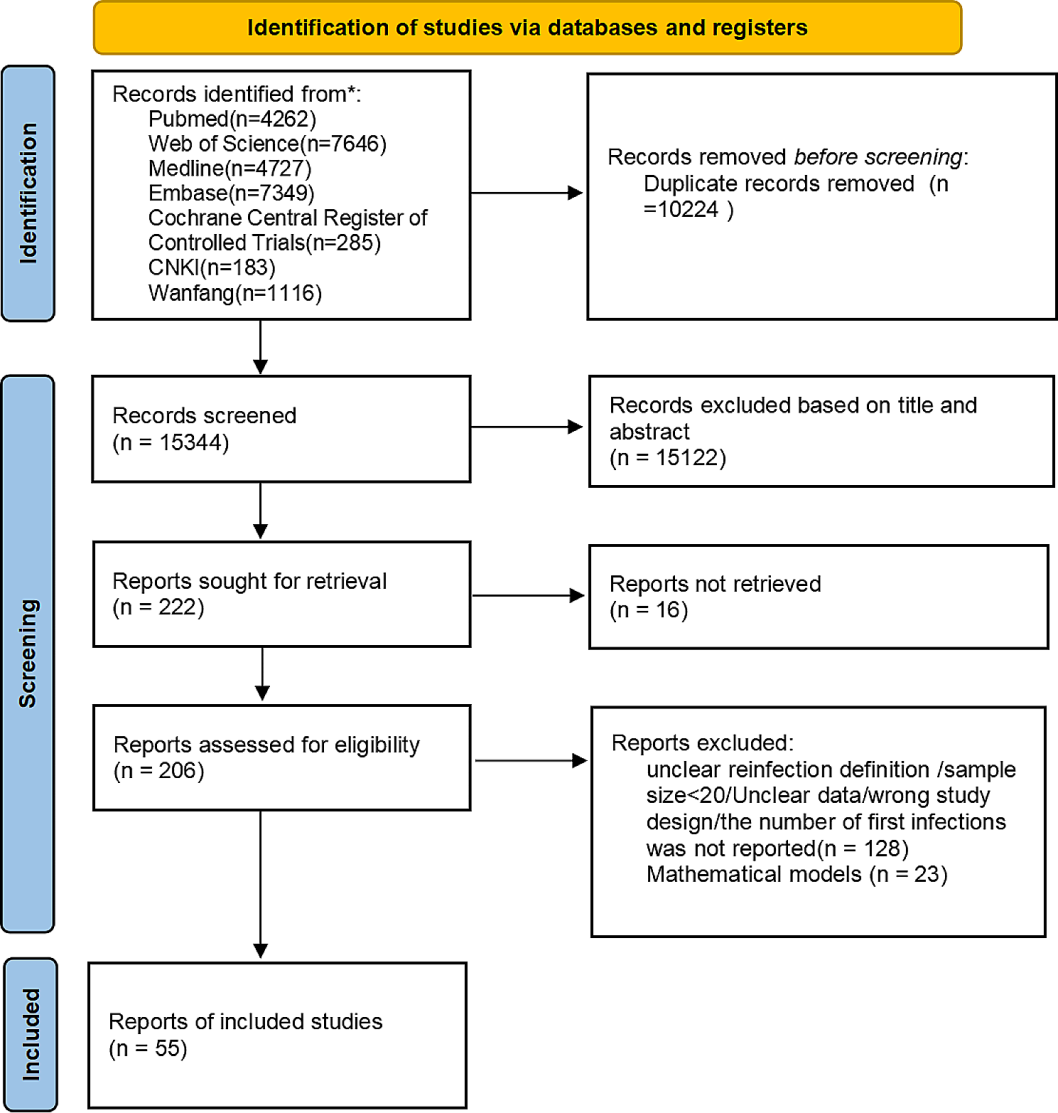
Flow chart of literature screening

By searching the above 7 databases, a total of 25,568 articles were retrieved, from which 10,224 duplicates were removed, resulting in 57 studies according to the inclusion and exclusion criteria. One of them reported only the reinfection rate, but not the associated number of first infections as well as the number of reinfections, and two other articles studied the same population, so the meta-analysis of single-group rates could not be performed. Therefore, we excluded the above literature with the number of unreported infections and selected one of two literature with the same population for inclusion. Finally, there were 55 studies included. The PRISMA flow chart was shown in Fig. 1. There were 46 cohort studies, 6 case-control studies, and 3 cross-sectional studies. For the cohort study, there were 2 studies on the risk of high bias. For case-control studies, there was no literature with a high risk of bias. For cross-sectional studies, there were 2 studies on the risk of high bias. Of the 55 included literature, 5 reported time-varying rates of infection and 19 reported variants of infection that either classified the reinfected population according to the time of reinfection. The research areas involved the United States, China, the United Kingdom, Italy, and so on.

### Subgroup analysis results of Meta-analysis

Through meta-analysis, a total of 14,681,235 subjects were included in the 55 studies, with a total of 111,846 cases of SARS-CoV-2 reinfection. The reported range of reinfection rate was between 0% and 28.4%, and the summarized reinfection rate was approximately 0.94% (95% *CI*: 0.65 -1.35%). The forest plot was shown in Additional file 6. Subgroup analyses were performed for age, reinfection variant, country, study population, and study type respectively, and the results were shown in Table 1, and forest plots for each subgroup analysis were shown in Additional file 6. After dividing the reinfection variants according to the time of reinfection reported in the literature, the reinfection rate of SARS-CoV-2 was 0.59% (95% *CI*: 0.43 -0.82%) for Alpha variants; 0.26% (95% *CI*: 0.10 -0.66%) for Wild variants; 0.41% (95% *CI*: 0.17 -0.97%) for Delta variants; 4.10% (95% *CI*: 1.36 -11.74%) for Omicron variants, and the difference in reinfection rate among different variants was statistically significant. Subgroup analysis of the different countries revealed that Brazil had the highest reinfection rate of 6.60% (95% *CI*: 3.94 -10.84%), Spain had a very low reinfection rate of 0.30% (95% *CI*: 0.05 -1.60%), and the combined reinfection rates in other countries were shown in Table 1. Subgroup analysis of the study types revealed that the SARS-CoV-2 reinfection rate in the cross-sectional study was 2.13% (95% *CI*: 1.14 -3.94%), with statistically significant differences from aggregated reinfection rates in other study types. Thus, reinfection variant, country, and study type may be significant influencing factors for study heterogeneity.

**Table 1:** Results of subgroup analysis

	Article number	Proportion	95% CI	τ^2^	*P*
**Age**					0.9317
>=18	4	0.0055	[0.0022; 0.0140]	0.8906	
< 18	2	0.0062	[0.0007; 0.0554]	2.5809	
**Reinfected variants**					**0.0016**
Wild	7	0.0026	[0.0010; 0.0066]	1.3937	
Alpha	2	0.0059	[0.0043; 0.0082]	0.0247	
Delta	3	0.0041	[0.0017; 0.0097]	0.5690	
Omicron	7	0.0410	[0.0136; 0.1174]	2.3479	
**Country**					**< 0.0001**
the United States	15	0.00106	[0.0051; 0.0222]	2.1674	
the United Kingdom	6	0.0048	[0.0037; 0.0064]	0.0819	
Türkiye	3	0.0064	[0.0015; 0.0266]	1.6089	
Italy	4	0.0119	[0.0033; 0.0416]	1.6623	
India	3	0.0297	[0.0031; 0.2332]	4.1048	
Iran	2	0.0147	[0.0043; 0.0490]	0.7578	
Israel	2	0.0147	[0.0121; 0.0179]	0.0196	
Spain	3	0.0030	[0.0005; 0.0160]	2.1176	
Canada	2	0.0136	[0.0134; 0.0139]	0	
Brazil	2	0.0660	[0.0394; 0.1084]	0.1224	
Denmark	2	0.0037	[0.0034; 0.0039]	0	
**Study population**					0.1131
general population	46	0.0081	[0.0056; 0.0119]	1.2939	
Healthcare workers	9	0.0192	[0.0071; 0.0508]	1.5238	
**Study type**					**0.0020**
cohort study	46	0.0097	[0.0064; 0.0147]	2.0886	
case-control study	6	0.0050	[0.0030; 0.0084]	0.3813	
cross-sectional study	3	0.0213	[0.0114; 0.0394]	0.3128	

Note: Bolded indicates *P* < 0.05

### Time-varying reinfection rate

**Fig. 2:**
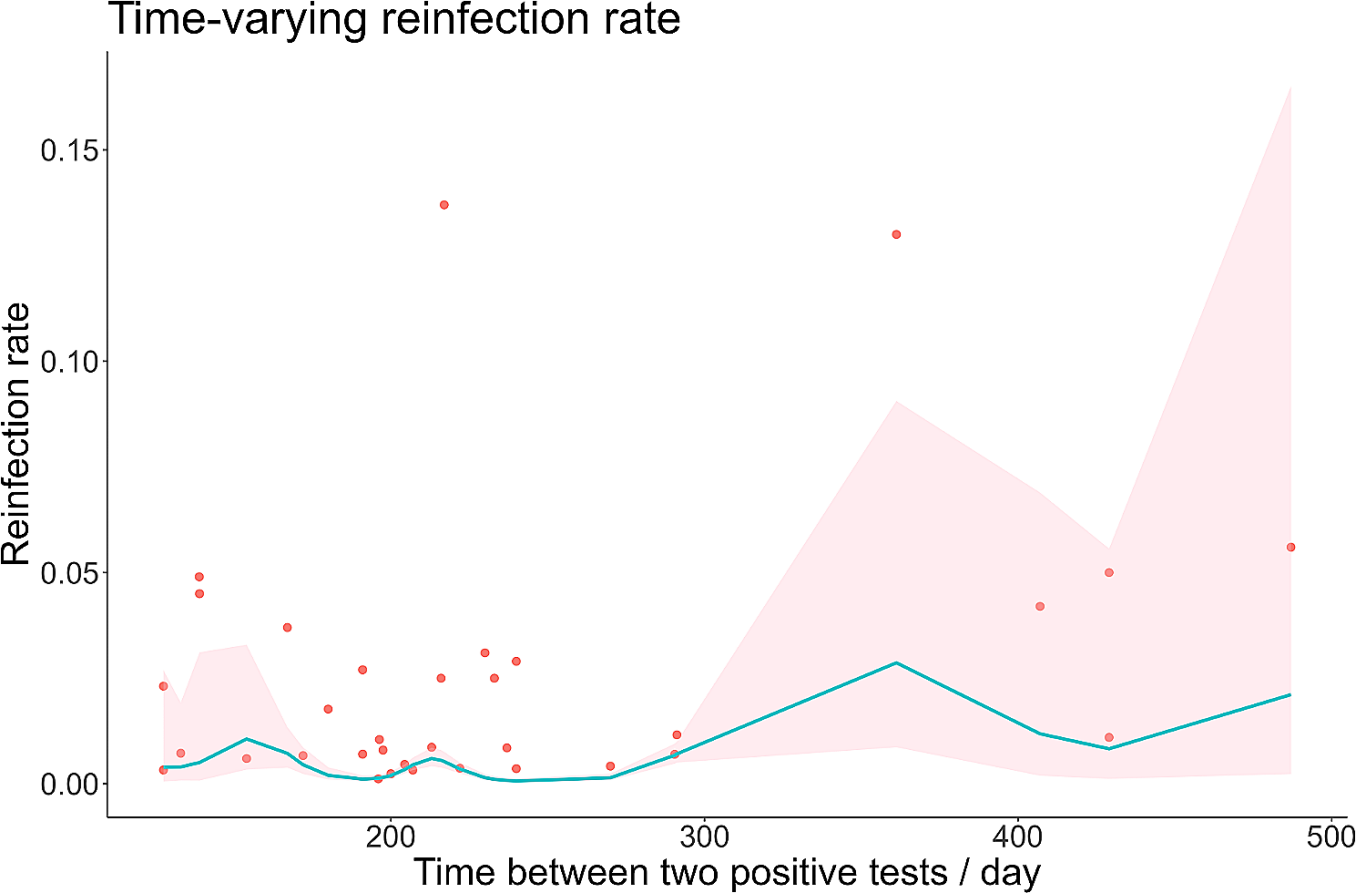
Meta-regression of time-varying reinfection rates (red dots indicate true values, shaded areas are 95% *CI*)

When estimating the time-varying reinfection rate, we took into account that it might not be possible to accurately determine whether the patients were reinfected or reinfected within a short time, so we only included literature with reinfection intervals > 90 days to estimate the time-varying reinfection rate. The meta-regression model for selecting non-stratified time-varying reinfection rates based on the Akaike information criterion(AIC) was presented in the form of splines with 8 degrees of freedom between reinfection interval and variant, and the calculation results of AIC were shown in Additional file 7. Based on the meta-regression results, we plotted the change curve of the reinfection rate concerning the reinfection interval, as shown in Fig. 2. The reinfection rate fluctuated with time, it first rose and then fell, and after a period of plateau then showed a trend of first rising and then falling again. The first inflection point was on day 154, with a predicted reinfection rate of 1.06% (95% *CI*: 0.34- 3.29%). The highest inflection point was on day 361, with a predicted reinfection rate of 2.86% (95% *CI*: 0.87- 9.06%). Although the second wave peak was lower than the first wave peak, the third wave peak was significantly higher than the first wave peak after a while. We speculated that one of the reasons why the third peak was higher than the first peak might be caused by the weakening of the population’s immunity level over time. This phenomenon might be due to the fact that immunization levels decreased over time after vaccination or infection with SARS-CoV-2. This was also consistent with other studies on antibody levels [24–26].

**Fig. 3:**
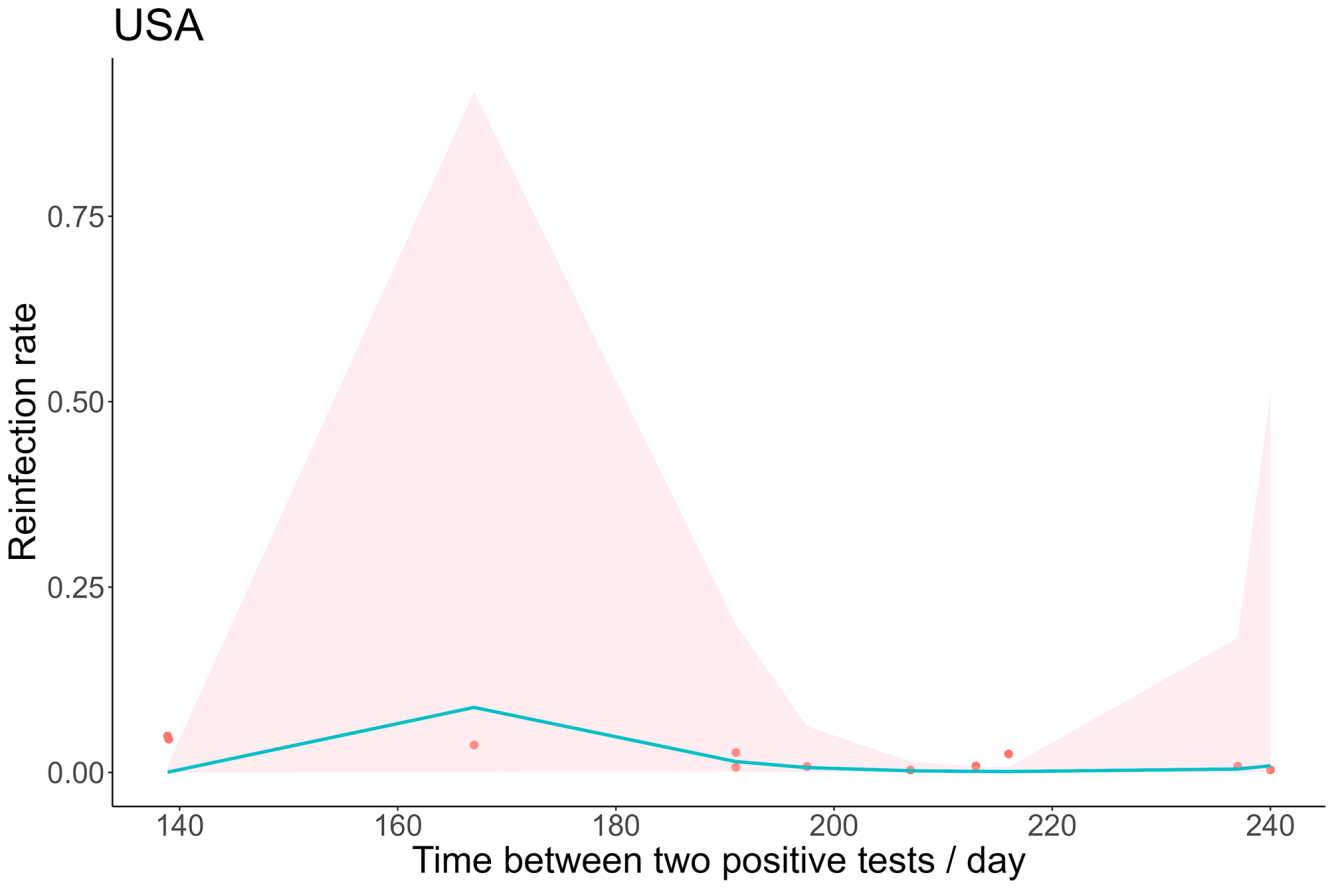
Meta-regression of time-varying reinfection rates in the United States (red dots indicate true values, shaded areas are 95% *CI*)

Since the quality of data in the United States is among the highest in the world, we also considered the trend of the SARS-CoV-2 reinfection rate in the United States over time. The reinfection rate increased first and then decreased with time in Fig. 3, and the peak was 0.23% (95% *CI*: 0.035-1.46%) on day 167, and the subsequent reinfection rate was no significant fluctuation.

**Fig. 4:**
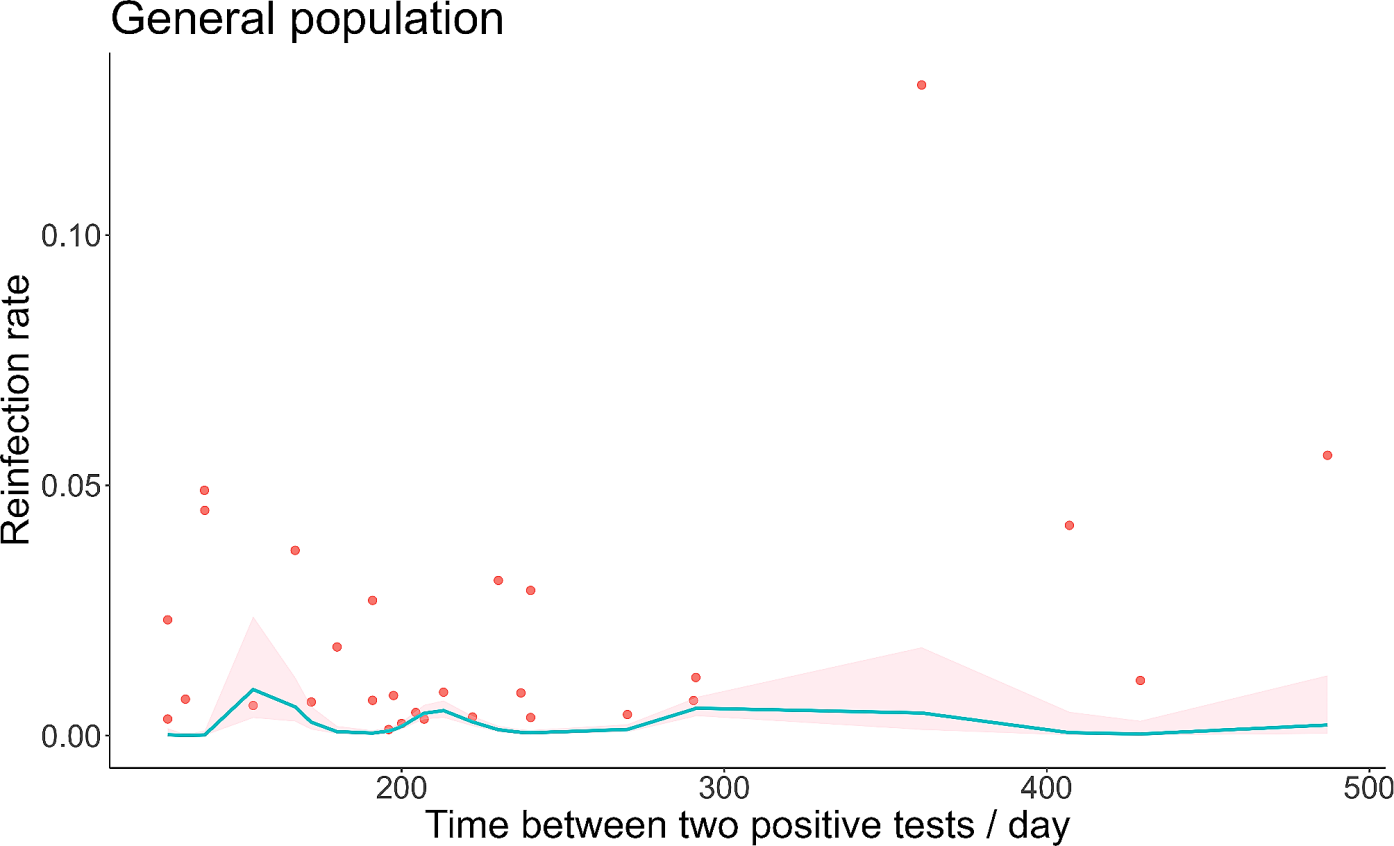
Meta-regression of time-varying reinfection rates among general population (red dots indicate true values, shaded areas are 95% *CI*)

**Fig. 5:**
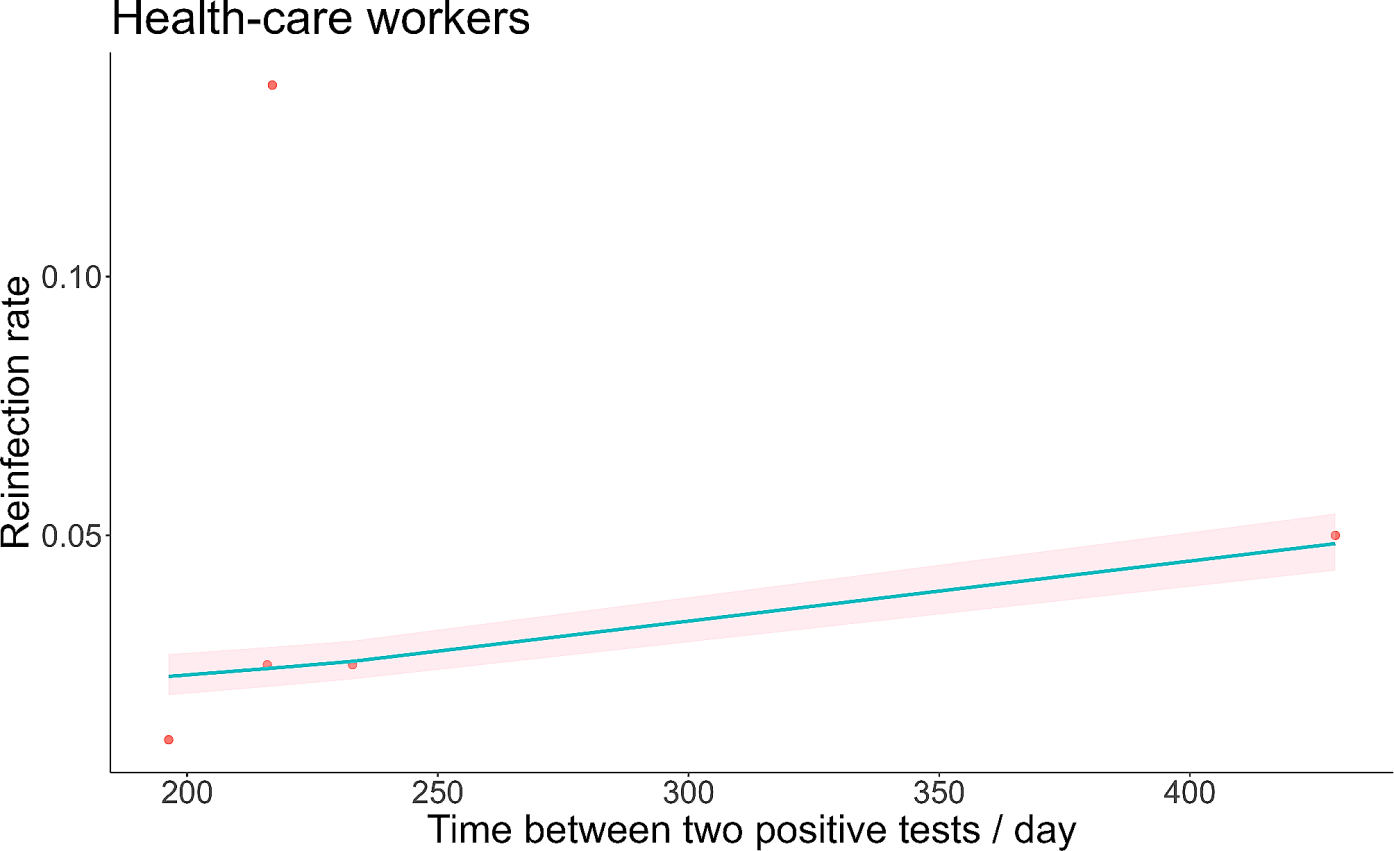
Meta-regression of time-varying reinfection rates among health care workers (red dots indicate true values, shaded areas are 95% *CI*)

The exposure patterns of the general population and healthcare workers were also different, so we analyzed the time-varying reinfection rates for these two groups separately, as shown in Figs. 4 and 5. The general population infection rate showed a trend of fluctuations, or after a period of time infection rose again. As shown in Fig. 4, there were roughly three peaks of reinfection in the general population, but the first peak was significantly larger than the last two peaks. The maximum was 0.92% (95% *CI*: 0.35 -2.38%) on day 154. The reinfection rate and 95% *CI* of healthcare workers showed an increasing trend over time. The maximum was 4.84% (95% *CI*: 4.32 -5.41%) on day 429.

### Sensitivity analysis

After excluding the literature with a high risk of bias, the combined SARS-CoV-2 reinfection rate was 0.82% (95% *CI*: 0.58 -1.17%), which was not significantly different from the aggregated SARS-CoV-2 reinfection rate before exclusion. Each included literature was excluded in turn, and the combined reinfection rate was recalculated. The results of the sensitivity analysis were shown in Additional file 8, and it was found that the exclusion of each literature did not significantly change the results of the study, and the change in reinfection rate ranged from 0.88 to 1.00%, which was more consistent with this meta-analysis of summarized reinfection.

### Spline regression of reinfection rate on time

In addition, we fitted the regression function between reinfection rate and reinfection interval by natural spline regression without considering the heterogeneity between the literature. The results were presented in Additional file 9. When fitted without subgroups, the reinfection rate increased first and then decreased, showing an ‘S’ pattern. Compared to the meta-regression result, the spline regression result was only one peak, with the time change trend more slowly.

Time-varying reinfection in the United States showed a decreasing trend over time. The maximum was 4.78% (95% *CI*: 3.18 -6.37%) on day 139 and the minimum was only 0.72% (95% *CI*: 0 -2.16%) on day 237. The time-varying reinfection rate in the cohort study showed an upward trend with a gradual increase in the rate of increase. Time-varying reinfection in the general population was the first to fall, then rose and then fell again in an ‘S’ pattern. The lowest peak of reinfection rate was 0.98% (95% *CI*: 0-2.19%) on day 200, and the highest peak of reinfection rate was 5.59% (95% *CI*: 2.98-8.20%) on day 429.

## Discussion

In this study, 55 articles related to SARS-CoV-2 reinfection were included for a meta-analysis of the global reinfection rate. The combined reinfection rate was found to be 0.94% (95% *CI*: 0.65 -1.35% ), with reinfection variant, country and study type potentially affecting the heterogeneity of reinfection rates in different studies. Furthermore, meta-regression was utilized to estimate the time-varying reinfection rate, revealing a fluctuating trend where the reinfection rate of SARS-CoV-2 would initially increase and then decrease, followed by an increase and another decline in a stable period. Sensitivity analysis demonstrated that the result of SARS-CoV-2 reinfection in our study was relatively stable at about 1%, regardless of whether the literature was excluded one by one or the literature with a high risk of bias. Therefore, we believe that the overall reinfection rate and the trend in reinfection rate obtained in this study can reflect the current global situation of SARS-CoV-2 reinfection.

The reinfection rate of SARS-CoV-2 in this study was 0.94% (95% *CI*: 0.65 -1.35%), slightly higher than that of Mao et al. [12], but lower than that of Ukwishaka et al. [27], which might be caused by the different research time range and the definition of reinfection in the literature included in this study. In this study, meta-regression was used to fit the trend of reinfection rate and the time interval between reinfection, and it was found that the reinfection rate showed a trend of fluctuations, which may be related to the weakening of the protective effect of previous infection and the protective effect of the vaccine against reinfection [28, 29]. We also observed similar trends in the general population subgroup analysis, namely, the reinfection rates again presented a downward trend after initially rising, but after a period of time,the reinfection rate might rise again. However, the meta-regression showed a continuous upward trend in reinfection rates over time for the general population subgroups. We suspected that the literature reported about the general population began in the early phase of the SARS-CoV-2 epidemic and thus reflects a gradual increase in reinfection rates. In contrast to meta-regression, the results of spline regression that did not account for heterogeneity showed an “S-shaped” trend in total reinfection and in the subgroup with the general population, while the United States showed subgroup showed an increased trend. The difference between the results of spline regression and meta-regression may be due to the fact that the spline regression did not take into account the effects of different countries and reinfection variants on the reinfection rate, while the subgroup analysis in meta-analysis has shown that country and reinfection strains are important factors affecting the pooled reinfection rate.

Any possible resurgence or vaccine breakthrough infection was not considered in this study. This study showed that the total reinfection rate varies among different reinfected variants, with the Omicron variant having a significantly higher reinfection rate than other variants. This is consistent with the research results of Ciuffreda [30], Cohen [31], and Pulliam JRC et al. [32]. The Omicron variant has a lower protective effect on reinfection compared to other variants, resulting in a significantly higher reinfection rate of the Omicron variant [33].

This study also indicated that the reinfection rate varies among different countries, such as Brazil, higher than other countries, with a reinfection rate of 6.60% (95% *CI*: 3.94 -10.84%), which migtht be influenced by different countries’ epidemic prevention policies, susceptible populations, ecological environment, etc. [34].

The search strategy we used was based on previous studies, and the process of literature screening and information extraction strictly followed PRISMA standards. In addition, the advantage of this study was that compared with the previous meta-analysis of the SARS-CoV-2 reinfection rate, first, to avoid the heterogeneous impact of SARS-CoV-2 reinfection definition on SARS-CoV-2 reinfection rates, so this study defined reinfection and included only literature with reinfection intervals greater than 90 days. Secondly, considering the heterogeneity of reinfection rates of different variants, we constructed a meta-regression of time-varying reinfection rates and compared it with the temporal spline regression without considering heterogeneity to explore the temporal trends of SARS-CoV-2 reinfection rates, which could provide a basis for subsequent research on prediction of SARS-CoV-2 infection, and could guide significance for the future policy formulation of SARS-CoV-2 reinfection. Our results warn us that the natural decay of immune levels over time may lead to the reinfection of SARS-CoV-2, resulting in a new round of COVID-19 pandemic. Moreover, the reinfection rate of SARS-CoV-2 varied greatly among different countries and under different variants. Therefore, to prevent the spread of SARS-CoV-2 and reinfection, countries with high reinfection rates in this study should predict the next epidemic peak based on the peak time of the previous epidemic wave and the peak time of reinfection pointed out in this study, to timely prepare epidemic materials and formulate relevant prevention and control policies to prevent the wide spread of SARS-CoV-2 infection. Further, we considered the results to be applied to the prediction of the SARS-CoV-2 epidemic model. For example, it is possible to add a time-varying reinfection rate to the classical transmission dynamics model based on meta-regression and spline regression. By combining real-world summary data with the mathematical model, we can predict the future incidence trend of SARS-CoV-2.

However, the limitations of this study are as follows: first, we did not include the “grey literature”, which may lack potential relevant studies that meet the inclusion criteria [35]. In addition, the pattern of publication bias in the field of single-arm study with single-group rate is not clear, and funnel plot analysis may lead to inaccurate conclusions. Second, there is still no clear global definition of SARS-CoV-2 reinfection. In the literature of reinfection searched in this study, most of the diagnostic criteria were at least 90 days between two positive laboratory tests. In order to avoid data heterogeneity in meta-analysis while ensuring data quantity, we used previous literature and previous definitions of reinfection in some countries as the criteria for included literature and data extraction in this study. Although we noted that clinical symptoms and real time-PCR or Rapid Antigen test were also important for the definition of SARS-CoV-2 reinfection, forming a globally harmonized standard definition was definitely beyond the scope of this study. Besides, due to the lack of specific data in the included literature, subgroup analysis of disease severity and vaccination could not be carried out, so it was impossible to explore all the sources of heterogeneity of SARS-CoV-2 reinfection rates. Meanwhile, the sample sizes of the literature included in this study were quite different, which might also be the source of heterogeneity in reinfection rates.

### Electronic supplementary material

Below is the link to the electronic supplementary material.

**Additional file 1:** PRISMA 2020 checklist.

**Additional file 2:** Search strategy.

**Additional file 3:** Information extraction form.

**Additional file 4:** Risk of bias assessments.

**Additional file 5:** Distribute transformation results.

**Additional file 6:** Meta-analysis results.

**Additional file 7:** The AIC of model.

**Additional file 8:** Sensitivity analysis results.

**Additional file 9:** Spline regression of reinfection rate.

## Data Availability

All data generated or analyzed during this study are included in this published article.

## Declarations

### Geolocation information

Our Geolocation information: Sichuan University Sect. 3. South Renmin Road, Wuhou, CHENGDU, China.

## References

[1] WHO. Coronavirus disease (COVID-19) pandemic. 2023. Available from: https://www.who.int/emergencies/diseases/novel-coronavirus-2019

[2] UN News. Available from: https://news.un.org/en/

[3] Msemburi W, Karlinsky A, Knutson V, Aleshin-Guendel S, Chatterji S, Wakefield J (2023). The WHO estimates of excess mortality associated with the COVID-19 pandemic. Nature.

[4] Guedes AR, Oliveira MS, Tavares BM, Luna-Muschi A, Lazari CDS, Montal AC (2023). Reinfection rate in a cohort of healthcare workers over 2 years of the COVID-19 pandemic. Sci Rep.

[5] Bastard J, Taisne B, Figoni J, Mailles A, Durand J, Fayad M (2022). Impact of the Omicron variant on SARS-CoV-2 reinfections in France, March 2021 to February 2022. Euro Surveill.

[6] Piazza MF, Amicizia D, Marchini F, Astengo M, Grammatico F, Battaglini A (2022). Who is at higher risk of SARS-CoV-2 reinfection? Results from a northern region of Italy. Vaccines (Basel).

[7] Babiker A, Marvil CE, Waggoner JJ, Collins MH, Piantadosi A (2021). The importance and challenges of identifying SARS-CoV-2 reinfections. J Clin Microbiol.

[8] Qureshi AI, Baskett WI, Huang W, Lobanova I, Hasan Naqvi S, Shyu CR (2022). Reinfection with severe acute respiratory syndrome coronavirus 2 (SARS-CoV-2) in patients undergoing serial laboratory testing. Clin Infect Dis.

[9] Arslan Y, Akgul F, Sevim B, Varol ZS, Tekin S (2022). Re-infection in COVID-19: do we exaggerate our worries?. Eur J Clin Invest.

[10] Ghorbani SS, Taherpour N, Bayat S, Ghajari H, Mohseni P, Nazari SSH (2022). Epidemiologic characteristics of cases with reinfection, recurrence, and hospital readmission due to COVID-19: a systematic review and meta‐analysis. J Med Virol.

[11] Nguyen NN, Nguyen YN, Hoang VT, Million M, Gautret P (2023). SARS-CoV-2 reinfection and severity of the disease: a systematic review and meta-analysis. Viruses.

[12] Mao Y, Wang W, Ma J, Wu S, Sun F (2021). Reinfection rates among patients previously infected by SARS-CoV-2: systematic review and meta-analysis. Chin Med J (Engl).

[13] To KK, Hung IF, Ip JD, Chu AW, Chan WM, Tam AR (2021). Coronavirus Disease 2019 (COVID-19) re-infection by a phylogenetically distinct severe acute respiratory syndrome coronavirus 2 strain confirmed by whole genome sequencing. Clin Infect Dis.

[14] Akkız H (2022). The biological functions and clinical significance of SARS-CoV-2 variants of corcern. Front Med (Lausanne).

[15] Pilz S, Theiler-Schwetz V, Trummer C, Krause R, Ioannidis JPA (2022). SARS-CoV-2 reinfections: overview of efficacy and duration of natural and hybrid immunity. Environ Res.

[16] Bowe B, Xie Y, Al-Aly Z (2022). Acute and postacute sequelae associated with SARS-CoV-2 reinfection. Nat Med.

[17] Graham MS, Sudre CH, May A, Antonelli M, Murray B, Varsavsky T (2021). Changes in symptomatology, reinfection, and transmissibility associated with the SARS-CoV-2 variant B.1.1.7: an ecological study. Lancet Public Health.

[18] Carazo S, Skowronski DM, Brisson M, Barkati S, Sauvageau C, Brousseau N (2023). Protection against omicron (B.1.1.529) BA.2 reinfection conferred by primary omicron BA.1 or pre-omicron SARS-CoV-2 infection among health-care workers with and without mRNA vaccination: a test-negative case-control study. Lancet Infect Dis.

[19] Finch E, Lowe R, Fischinger S, de St Aubin M, Siddiqui SM, Dayal D (2022). SARS-CoV-2 antibodies protect against reinfection for at least 6 months in a multicentre seroepidemiological workplace cohort. PLoS Biol.

[20] Euser SM, Weenink T, Prins JM, Haverkort M, Manders I, van Lelyveld S (2022). The effect of varying interval definitions on the prevalence of SARS-CoV-2 reinfections: a retrospective cross-sectional cohort study. Diagnostics (Basel).

[21] Public health surveillance for COVID-19. interim guidance. Available from:https://www.who.int/publications-detail-redirect/WHO-2019-nCoV-SurveillanceGuidance-2022.2

[22] CDC. COVID-19 and Your Health. Available from:https://www.cdc.gov/coronavirus/2019-ncov/your-health/reinfection.html

[23] Ho P, Bulsara M, Downs J, Patman S, Bulsara C, Hill AM (2019). Incidence and prevalence of falls in adults with intellectual disability living in the community: a systematic review. JBI Database Syst Rev Implement Rep.

[24] Israel A, Shenhar Y, Green I, Merzon E, Golan-Cohen A, Ruppin E (2021). Large-scale study of antibody Titer Decay following BNT162b2 mRNA vaccine or SARS-CoV-2 infection. Vaccines.

[25] Gaebler C, Wang Z, Lorenzi JCC, Muecksch F, Finkin S, Tokuyama M (2021). Evolution of antibody immunity to SARS-CoV-2. Nature.

[26] Wheatley AK, Juno JA, Wang JJ, Selva KJ, Reynaldi A, Tan HX (2021). Evolution of immune responses to SARS-CoV-2 in mild-moderate COVID-19. Nat Commun.

[27] Hooi JKY, Lai WY, Ng WK, Suen MMY, Underwood FE, Tanyingoh D (2017). Global prevalence of helicobacter pylori infection: systematic review and meta-analysis. Gastroenterology.

[28] Stein C, Nassereldine H, Sorensen RJD, Amlag JO, Bisignano C, Byrne S (2023). Past SARS-CoV-2 infection protection against re-infection: a systematic review and meta-analysis. Lancet.

[29] Yang H, Xie Y, Li C (2023). Understanding the mechanisms for COVID-19 vaccine’s protection against infection and severe disease. Expert Rev Vaccines.

[30] Ciuffreda L, Lorenzo-Salazar JM, García-Martínez de Artola D, Gil-Campesino H, Alcoba-Florez J, Rodríguez-Pérez H (2023). Reinfection rate and disease severity of the BA.5 omicron SARS-CoV-2 lineage compared to previously circulating variants of concern in the Canary Islands (Spain). Emerg Microbes Infect.

[31] Cohen D, Izak M, Stoyanov E, Mandelboim M, Perlman S, Amir Y (2023). Predictors of reinfection with pre-omicron and omicron variants of concern among individuals who recovered from COVID-19 in the first year of the pandemic. Int J Infect Dis.

[32] Pulliam JRC, van Schalkwyk C, Govender N, von Gottberg A, Cohen C, Groome MJ (2022). Increased risk of SARS-CoV-2 reinfection associated with emergence of omicron in South Africa. Science.

[33] Bobrovitz N, Ware H, Ma X, Li Z, Hosseini R, Cao C (2023). Protective effectiveness of previous SARS-CoV-2 infection and hybrid immunity against the omicron variant and severe disease: a systematic review and meta-regression. Lancet Infect Dis.

[34] Deng L, Li P, Zhang X, Jiang Q, Turner D, Zhou C (2022). Risk of SARS-CoV-2 reinfection: a systematic review and meta-analysis. Sci Rep.

[35] Dillner P, Eggenschwiler LC, Rutjes AWS, Berg L, Musy SN, Simon M (2023). Incidence and characteristics of adverse events in paediatric inpatient care: a systematic review and meta-analysis. BMJ Qual Saf.

