# Supplementary material for: How does the SARS-CoV-2 reinfection rate change over time? The global evidence from systematic review and meta-analysis
Source: BMC Infect Dis. 2024 Mar 21;24:339. doi: 10.1186/s12879-024-09225-z (PMC10956270; doi:10.1186/s12879-024-09225-z)
Supplement: Supplementary file 2 — Additional file 2: Search strategy. [file 12879_2024_9225_MOESM2_ESM.docx]

**Additional file 2. Search strategy**

The search strategy includes two core terms, SARS-CoV-2 and reinfection. Moreover, these two keywords are extended to include words with similar meanings to them. It is mainly searched by the search strategy of “keyword A or other free words” and “keyword B or other free words”. The study searched the literature in Pubmed, Web of Science, Medline (Ovid), Embase (Ovid), Cochrane Central Register of Controlled Trials, China National Knowledge Infrastructure (CNKI), Wanfang from January 1, 2020 to March 16, 2023. The results of each search library were imported into Endnote X9 in RIS format, and the duplicate literature was screened out using Endnote X9.

**1 Pubmed**

| Search number | Query | Results |
| --- | --- | --- |
| 11 | #5 AND #10 | 4,262 |
| 10 | #6 OR #7 OR #8 OR #9 | 394,880 |
| 9 | ("repeat positive"[Title/Abstract] OR "re-positive"[Title/Abstract] OR "two positive"[Title/Abstract] OR "2 positive"[Title/Abstract]) AND ("pcr"[Title/Abstract] OR "polymerase chain reaction"[Title/Abstract]) | 2,055 |
| 8 | "repeat infect*"[Title/Abstract] OR "second infect*"[Title/Abstract] OR ("reactivat*"[All Fields] AND "infect*"[Title/Abstract]) OR "recurrent infect*"[Title/Abstract] OR "double infect*"[Title/Abstract] OR "multiple infect*"[Title/Abstract] | 30,345 |
| 7 | "reinfect*"[Title/Abstract] OR "re infect*"[Title/Abstract] OR "relapse*"[Title/Abstract] | 205,825 |
| 6 | "reinfection"[MeSH Terms] OR "recurrence"[MeSH Terms] | 199,507 |
| 5 | #1 OR #2 OR #3 OR #4 | 344,353 |
| 4 | ("coronavirus*"[Title/Abstract] OR "corona virus*"[Title/Abstract] OR "betacoronavirus*"[Title/Abstract]) AND ("pandemic*"[Title/Abstract] OR "epidemic*"[Title/Abstract] OR "outbreak*"[Title/Abstract] OR "crisis"[Title/Abstract]) | 69,513 |
| 3 | ("coronavirus*"[Title/Abstract] OR "corona virus*"[Title/Abstract] OR "betacoronavirus*"[Title/Abstract]) AND ("new"[Title/Abstract] OR "novel"[Title/Abstract] OR "19"[Title/Abstract] OR "2019"[Title/Abstract] OR "Wuhan"[Title/Abstract] OR "Hubei"[Title/Abstract]) | 112,465 |
| 2 | "SARS-CoV-2"[MeSH Terms] OR "SARS-CoV-2"[Title/Abstract] OR "SARSCoV2"[Title/Abstract] OR "SARSCoV-2"[Title/Abstract] OR "SARS-CoV2"[Title/Abstract] OR "sars coronavirus 2"[Title/Abstract] OR "severe acute respiratory syndrome coronavirus 2"[Title/Abstract] OR "severe acute respiratory syndrome corona virus 2"[Title/Abstract] | 197,566 |
| 1 | "COVID-19"[MeSH Terms] OR "COVID-19"[Title/Abstract] OR "COVID19"[Title/Abstract] OR "2019-nCoV"[Title/Abstract] OR "2019nCoV"[Title/Abstract] OR "nCOV-19"[Title/Abstract] OR "nCOV19"[Title/Abstract] | 327,558 |

**2 Web of Science**

(Editions:WOS.IC,WOS.CCR,WOS.SCI,WOS.AHCI,WOS.ESCI,WOS.ISTP,WOS.SSCI,WOS.ISSHP,INSPEC.INSPEC,KJD.KJD,MEDLINE.MEDLINE,SCIELO.SCIELO)

| # | Search Query | Results |
| --- | --- | --- |
| 1 | TS=(COVID-19 or COVID19 or 2019-nCoV or 2019nCoV or nCOV-19 or nCOV19 or SARS-COV-2 or SARSCOV-2 or SARSCOV2 or SARS-COV2 or SARS coronavirus 2 or Severe Acute Respiratory Syndrome Coronavirus 2 or Severe Acute Respiratory Syndrome Corona Virus 2) | 506567 |
| 2 | TS=((new or novel or "19" or "2019" or Wuhan or Hubei) NEAR/3 (coronavirus* or "corona virus*" or betacoronavirus*)) | 183086 |
| 3 | TS=((coronavirus* or "corona virus*" or betacoronavirus*) NEAR/3 (pandemic* or epidemic* or outbreak* or crisis)) | 29224 |
| 4 | #1 OR #2 OR #3 | 513209 |
| 5 | TS=(reinfect* or re-infect* or relapse* or recurren*) | 1348602 |
| 6 | TS=((repeat* or second* or reactivat* or recurrent* or double or multiple) NEAR/2 (infect*)) | 98784 |
| 7 | TS=(("repeat positive" or "re-positive" or "two positive" or "2 positive") NEAR/5 (pcr or “polymerase chain reaction”)) | 395 |
| 8 | #6 OR #7 OR #5 | 1415886 |
| 9 | #4 AND #8 and 2020 or 2021 or 2022 or 2023 (Publication Years) and Case Report or Book or News or Letter or Abstract or Reference Material or Report (Exclude – Document Types) | 7646 |

**3 Ovid MEDLINE(R) ALL <1946 to March 09, 2023>**

| # | Query | Results from 10 Mar 2023 |
| --- | --- | --- |
| 1 | exp COVID-19/ | 212,858 |
| 2 | exp SARS-CoV-2/ | 149,409 |
| 3 | (COVID-19 or COVID19 or 2019-nCoV or 2019nCoV or nCOV-19 or nCOV19).ti,ab,kf,ot. | 303,121 |
| 4 | (SARS-COV-2 or SARSCOV-2 or SARS-COV2 or SARSCOV2 or SARS coronavirus 2 or Severe Acute Respiratory Syndrome Coronavirus 2 or Severe Acute Respiratory Syndrome Corona Virus 2).ti,ab,kf,ot. | 118,474 |
| 5 | ((Coronavirus* or Corona virus* or betacoronavirus*) adj3 (new or novel or "19" or "2019" or Wuhan or Hubei)).ti,ab,kf,ot. | 82,385 |
| 6 | ((coronavirus* or corona virus* or betacoronavirus*) adj3 (pandemic* or epidemic* or outbreak* or crisis)).ti,ab,kf,ot. | 15,068 |
| 7 | 1 or 2 or 3 or 4 or 5 or 6 | 342,064 |
| 8 | exp Reinfection/ | 918 |
| 9 | exp Recurrence/ | 199,554 |
| 10 | (reinfect* or re-infect* or relapse*).ti,ab,kf,ot. | 205,820 |
| 11 | ((repeat* or second* or reactivat* or recurrent* or double or multiple) adj2 infect*).ti,ab,kf,ot. | 44,474 |
| 12 | (("repeat positive" or "re-positive" or "two positive" or "2 positive") adj5 (pcr or polymerase chain reaction)).ti,ab,kf,ot. | 272 |
| 13 | 8 or 9 or 10 or 11 or 12 | 406,274 |
| 14 | 7 and 13 | 4,727 |

**4 Embase <1974 to 2023 March 09>**

| # | Query | Results from 10 Mar 2023 |
| --- | --- | --- |
| 1 | exp coronavirus disease 2019/ | 339,709 |
| 2 | exp Severe acute respiratory syndrome coronavirus 2/ | 94,902 |
| 3 | exp asymptomatic coronavirus disease 2019/ | 2,065 |
| 4 | (COVID-19 or COVID19 or 2019-nCoV or 2019nCoV or nCOV-19 or nCOV19).ti,ab,kf,ot. | 367,737 |
| 5 | (SARS-COV-2 or SARSCOV-2 or SARS-COV2 or SARSCOV2 or SARS coronavirus 2 or Severe Acute Respiratory Syndrome Coronavirus 2 or Severe Acute Respiratory Syndrome Corona Virus 2).ti,ab,kf,ot. | 147,725 |
| 6 | ((Coronavirus* or Corona virus* or betacoronavirus*) adj3 (new or novel or "19" or "2019" or Wuhan or Hubei)).ti,ab,kf,ot. | 92,919 |
| 7 | ((coronavirus* or corona virus* or betacoronavirus*) adj3 (pandemic* or epidemic* or outbreak* or crisis)).ti,ab,kf,ot. | 16,557 |
| 8 | 1 or 2 or 3 or 4 or 5 or 6 or 7 | 435,988 |
| 9 | exp reinfection/ | 34,334 |
| 10 | exp recurrent infection/ | 22,501 |
| 11 | (reinfect* or re-infect* or relapse*).ti,ab,kf,ot. | 363,692 |
| 12 | ((repeat* or second* or reactivat* or recurrent* or double or multiple) adj2 infect*).ti,ab,kf,ot. | 63,825 |
| 13 | (("repeat positive" or "re-positive" or "two positive" or "2 positive") adj5 (pcr or polymerase chain reaction)).ti,ab,kf,ot. | 433 |
| 14 | 9 or 10 or 11 or 12 or 13 | 440,533 |
| 15 | 8 and 14 | 7,349 |

**5 CNKI**

Abstract: SARS-CoV-2 and Abstract: (reinfection + superinfection)

**6 WanFang**

Subject: SARS-CoV-2 and Subject: (reinfection + superinfection)

**7 Cochrane Central Register of Controlled Trials**

| ID | Search | Hits |
| --- | --- | --- |
| #1 | MeSH descriptor: [COVID-19] explode all trees | 3974 |
| #2 | MeSH descriptor: [SARS-CoV-2] explode all trees | 2174 |
| #3 | COVID-19 or COVID19 or "2019-nCoV" or 2019nCoV or "nCOV-19" or nCOV19 or SARS-COV-2 or SARSCOV-2 or SARS-COV2 or SARSCOV2 or SARS coronavirus 2 or Severe Acute Respiratory Syndrome Coronavirus 2 or Severe Acute Respiratory Syndrome Corona Virus 2.tw,kw. | 15202 |
| #4 | (new or novel or "19" or "2019" or Wuhan or Hubei) adj3 (coronavirus* or corona virus* or betacoronavirus*).tw,kw. | 219 |
| #5 | (coronavirus* or corona virus* or betacoronavirus*) adj3 (pandemic* or epidemic* or outbreak* or crisis).tw,kw. | 209 |
| #6 | #1 or #2 or #3 or #4 or #5 | 15362 |
| #7 | MeSH descriptor: [Reinfection] explode all trees | 17 |
| #8 | MeSH descriptor: [Recurrence] explode all trees | 14408 |
| #9 | reinfect* or re-infect* or replase*.tw,kw | 1462 |
| #10 | (repeat* or second* or reactivat* or recurrent* or double or multiple) adj2 (infect*).tw,kw. | 1143 |
| #11 | ("repeat positive" or "re-positive" or "two positive" or "2 positive") adj5 (pcr or polymerase chain reaction).tw,kw. | 169 |
| #12 | #7 or #8 or #9 or #10 or #11 | 16748 |
| #13 | #6 and #12 | 285 |
