# Supplementary material for: How does the SARS-CoV-2 reinfection rate change over time? The global evidence from systematic review and meta-analysis
Source: BMC Infect Dis. 2024 Mar 21;24:339. doi: 10.1186/s12879-024-09225-z (PMC10956270; doi:10.1186/s12879-024-09225-z)
Supplement: Supplementary file 3 — Additional file 3: Information extraction form. [file 12879_2024_9225_MOESM3_ESM.docx]

**Additional file 3. Information extraction form**

We used EXCEL software for information extraction, which was completed by two independent reviewers(CY, WZH, HXY, CMS, LX, HHP), and the conflict part was judged by a third party(CY, WZH). We defined the reinfection rate as the ratio of the number of reinfections to the number of first infections among the number of first infections. We not only collected the overall COVID-19 reinfection rate of the study, but also collected the reinfection rate of different ages, different strains that may be mentioned in the study.

| First author | Study period | Country | Study population | Number | Study design | Time-differentiated reinfected strains | Time between two positive tests (days) | Age | Reinfection rate | Number of reinfections |
| --- | --- | --- | --- | --- | --- | --- | --- | --- | --- | --- |
| Benjamin Bowe[1] | NA | America | general population | 5819264 | case-control study | delta and omicron | Median (IQR) :191(127-330) days | All ages | 0.007036 | 40947 |
| Anna Jeffery-Smith[2] | May 2020 to October 2020 | United Kingdom | general population | 88 | cohort study | wild type (dominates); alpha | NA | All ages | 0.011 | 1 |
| Josè Vitale[3] | February 2020 to February 28, 2021 | Italy | general population | 1579 | case-control study | wild type; alpha | Mean (SD): 230 (90) days | All ages | 0.031 | 5 |
| Mark S Graham[4] | September 28 to December 27, 2020 | United Kingdom | general population | 36509 | cohort study | alpha | ≥90天 | All ages |  | 249 |
| Luis Pampa-Espinoza[5] | April 2020 to May 2021 | Peru | general population | 1695 | cohort study | lambda (dominates), gamma | ≥6 months | All ages | 0.0177 | 30 |
| Sumit Malhotra[6] | February 1, 2022 to February 25, 2022 | India | Health-care workers | 3545 | cohort study | omicron | NA | All ages | 0.284 | 1007 |
| Philippe Brouqui[7] | January 27, 2020 to January 12, 2021 | France | general population | 6771 | cohort study | wild type; alpha; beta; | Mean: 172 (90-308) days | All ages | 0.0067 | 46 |
| Godwin E Akpan[8] | March 2020 to July 2021 | Liberia | general population | 5459 | cohort study | wild type; alpha; beta; delta | Median (IQR) :200 (99-415) days | 21-74 | 0.0024 | 13 |
| Fariba Zare[9] | March 20, 2020 to November 20, 2020 | Iran | general population | 4039 | cohort study | wild type; beta | 134.4±64.5 days | All ages | 0.0025 | 10 |
| Naila A Shaheen[10] | March 2020 to August 2021 | Saudi Arabia | general population | 35288 | cohort study | wild type; alpha; beta; delta | Median (IQR) :222 (90-462) days | All ages | 0.0037 | 132 |
| Anna A Mensah[11] | January 2020 to early May 2021 | United Kingdom | general population | 3860054 | case-control study | wild type; alpha; delta | NA | All ages | 0.0036 | 13960 |
| Hiam Chemaitelly[12] | February 28, 2020 to June 5, 2022 | Dubai | general population | 301943 | cohort study | wild type; alpha; beta; gamma; delta; omicron | Median (IQR) :154 (65-224) days | All ages | 0.006 | 1806 |
| Annalisa Quattrocchi[13] | June to August 2021 | the Republic of Cyprus | general population | 44227 | case-control study | delta | NA | >=18 | 0.002 | 93 |
| Nickolas Lewis[14] | March 1, 2020 to January 2022 | America | general population | 100517;  According to the strain: wild type period: 100517;  Alpha period: 100517;  Delta period: 100517 | cohort study | delta; omicron | Median (IQR): 7.9(4.8-10.7) months | >=12 | 0.0204642; According to the strain:wild type period: 0.00851597;  Alpha period: 0.00329298;  Delta period: 0.00865525 | 2057;  According to the strain: wild type period: 856;  Alpha period: 331;  Delta period: 870 |
| Ariel Hammerman[15] | August 23, 2020 to November 26, 2021 | Israel | Health-care workers | 149032 | cohort study | alpha; delta | NA | All ages | 0.01692254 | 2522 |
| Oriol Yuguero[16] | March 1 to November 30, 2020 | Spain | general population | 27758 | cohort study | wild type (dominates); alpha | NA | All ages | 0.0005 | 14 |
| Sara Carazo[17] | December 26, 2021 to March 12, 2022 | Canada | general population | 696439 | case-control study | omicron | Median (IQR) :407 (354-480) days | >=12 | 0.042 | 9505 |
| A. de Arriba Fernández[18] | June 1, 2021 to February 28, 2022 | Spain | general population | 110726 | cohort study | alpha; delta | NA | >=12 | 0.0031 | 340 |
| İrem Ceren Erbaş[19] | March 2020 to July 2021 | Türkiye | general population | 8840 | cohort study | wild type; alpha; beta; delta | Median (IQR) :196 (92-483) days | <18 | 0.0012 | 11 |
| Valentina Pecoraro[20] | January 1, 2021 to June 30, 2021 | Italy | general population | 0-14 year:4926；15-29year: 6347；30-49 year: 10166；50-69 year: 9488；>70 year: 4765 | cross-sectional study | alpha; gamma; delta; omicron | Mean: 313 days | All ages | 0-14 year:0.0042；15-29 year:0.0040；30-49 year:0.0040；50-69 year:0.0026；>70 year:0.0029 | 0-14 year:208；15-29 year:252；30-49 year:414；50-69 year:246；>70 year:138 |
| Jonathan Bastard[21] | January 1, 2021 to February 20, 2022 | France | general population | 18661139 | cross-sectional study | omicron | Mean: 244 days; Median (IQR): 267 (166-314) days | All ages | 0.031 | 584129 |
| J. Richards[22] | March 1, 2020 to January 10, 2021 | America | general population | 2625 | cohort study | wild type; alpha | Median(IQR) :126.50 (105.50，171.00) days | All ages | 0.059 | 156 |
| S. Medic[23] | March 6, 2020 to the end of July 2022 | Serbia | general population | 32524 | cohort study | alpha; beta; delta | Mean (SD): 240(117) days | <18 | 0.029 | 964 |
| Ferhat Arslan[24] | March 2020 to May 2021 | Istanbul | general population | 32607 | cohort study | wild type; alpha; beta; delta | NA | 17-71 | 0.0008 | 27 |
| Sharon M. Casey[25] | January 1, 2020 to February 28, 2021 | America | general population | 2431 | cohort study | alpha; gamma | Mean (SD): 191(65) days | All ages | 0.027 | 65 |
| B. L. Hønge[26] | February 2020 to August 2021 | Denmark | general population | 3806 | cohort study | wild type; alpha; delta | NA | 17-69 | 0.006 | 21 |
| Daniela Michlmayr[27] | February 1, 2020 to June 30, 2021 | Denmark | general population | 198817 | cohort study | alpha; delta; omicron | NA | >=2 | 0.1252 | 720 |
| Carlota Dobaño[28] | March 2020 to April 2021 | Spain | general population | 173 | cohort study | wild type; alpha | Mean: 4.25 months | All ages | 0.023121 | 4 |
| Michael B Rothberg[29] | March 9, 2020 to March 1, 2022 | America | general population | 635341 | cohort study | omicron | 398.7± 124.8 days | All ages |  | 1467 |
| Joanne Lacy[30] | March 1, 2020 to December 31, 2020 | United Kingdom | general population | 517870 | cohort study | wild type; alpha | NA | >=10 | 0.005435727 | 2815 |
| Jeff Slezak[31] | March 1, 2020 to October 31, 2020 | 南加州 | general population | 75149 | cohort study | wild type | ≤270 days | All ages | 0.0042 | 315 |
| M E Flacco[32] | March 3, 2020 to May 21, 2021 | Italy | general population | 7173 | cohort study | wild type; alpha; gamma | Mean (SD): 201(61) days | All ages | 0.0033 | 34 |
| Isabel Cristina Hurtado[33] | March 9, 2020 to June 30, 2021 | Colombia | general population | 327886 | cross-sectional study | wild type; alpha; gamma | Median: 182 (IC 95%: 90-154) days | All ages | 0.011 | 3249 |
| Carlos A Prete Jr[34] | April 1, 2020 to 2021 | Brazil | general population | 238 | cohort study | gamma | NA | All ages | 0.1008 | 24 |
| Yusuf Arslan[35] | March 11, 2020 to August 31, 2021 | Türkiye | general population | 58811 | cohort study | wild type; alpha; beta; delta | Median: 290.5±105.3 days | All ages | 0.007 | 421 |
| Ana Rubia Guedes[36] | March 10, 2020 to March 10, 2022 | Brazil | Health-care workers | 5865 | cohort study | omicron | Mean: 429(122-674) days | All ages | 0.05 | 284 |
| megan M[37] | March 12, 2020 to August 30, 2020 | America | general population | 150325 | cohort study | wild type | 138.9 ± 46.3 days | All ages | 0.049 | 63 |
| Anna Jeffery-Smith[38] | April 10, 2020 to January 31, 2021 | United Kingdom | general population | 1377 | cohort study | wild type; alpha | Mean: 133(86-161) days | 20-99 | 0.007262164 | 10 |
| Adnan I Qureshi[39] | December 1, 2019 to November 13, 2020 | America | general population | 9119 | cohort study | wild type | Mean(±SD): 116±21 days | All ages | 0.007 | 63 |
| Lara J Akinbami[40] | May 2020 to August 2020 | America | Health-care workers | 1572 | cohort study | wild type; alpha | Mean: 216.0(95% CI, 198.8-233.1) days | All ages | 0.025 | 40 |
| Masoud Alebouyeh[41] | March 2020 to March 2021 | Iran | Health-care workers | 490 | cohort study | wild type; alpha; beta; gamma; delta | 74-360 days | All ages | 0.137 | 18 |
| Mahdi Barzegar[42] | January 1, 2020 to August 22, 2021 | Iran | general population | 6240 | case-control study | wild type; alpha; beta; delta | NA | All ages | NA | NA |
| Antonio Leidi[43] | April 2020 to January 2021 | Switzerland | general population | 498 | cohort study | wild type | NA | >=12 | 0.01 | 5 |
| Anna A Mensah[44] | January 2020 to July 2021 | United Kingdom | general population | 688418 | cohort study | wild type; alpha; delta | NA | <16 | 0.0068 | 2343 |
| Osman Özüdoğru[45] | April 22, 2021 to January 26, 2022 | Türkiye | general population | 27487;  According to the strain: Alpha(5554)；Delta(17941)；Omicron(3992) | cross-sectional study | alpha; delta; omicron | 361.2±131.6 days | All ages | 0.027;  According to the strain: Alpha(0.46%)；Delta(1.16%)；Omicron(13.0%) | 755;  According to the strain:Alpha(24)；Delta(209)；Omicron(520) |
| Sumit Malhotra[46] | March 3, 2020 to June 18, 2021 | India | Health-care workers | 4953 | cohort study | wild type; alpha; delta | Median (IQR): 233(175-321) days | All ages | 0.025 | 124 |
| John T. Wilkins[47] | May 26 to January 8, 2021 | America | Health-care workers | 4947 | cohort study | delta; omicron | NA | >=18 | 0 | 8 |
| Mary K Good[48] | January to November 2021 | America | general population | 1378 | cohort study | wild type; alpha; gamma; delta | Mean：197.5(95-301) days | 18-27 | 0.008 | 11 |
| David J Bean[49] | March 12, 2020 to January 21, 2021 | America | general population | 1669 | cohort study | wild type; alpha | Median: 139(91-298) days | All ages | 0.045 | 75 |
| Eric Ochoa-Hein[50] | March 2020 to February 2022 | Mexico | general population | 1388 | cohort study | omicron | Median: 487(99-664) days | All ages | 0.056 | 73 |
| Wataru Ando[51] | January 1, 2020 to August 27, 2021 | America | general population | 165320 | cohort study | wild type; alpha; gamma; delta | Median: 167(122-230) days | All ages | 0.037 | 6133 |
| Alexander Lawandi[52] | June 1, 2020 to February 28, 2021 | America | general population | 51484 | cohort study | wild type; alpha | NA | All ages | 0.1 | 253 |
| S. Gazit[53] | March 1, 2020 to December 13, 2021 | Israel | general population | 107413 | cohort study | alpha; delta (dominates) | NA | >=16 | 0.013 | 1374 |
| Étienne Racine[54] | August 21, 2020 to March 1, 2022 | Canada | Health-care workers | 569 | cohort study | wild type; gamma; delta; omicron | Mean: 196.33 days | 18-75 | 0.0105 | 6 |
| Maria Francesca Piazza[55] | September 2021 to May 2022 | Italy | general population | 335117 | cohort study | delta\omicron (dominates) | NA | All ages | 0.05 | 15795 |

If the paper did not mention the reinfection variant, we classified the reinfection variants based on the WHO standards according to the study period. The discovery times of different variants are shown in Figure 3-1.The epidemic situation of SARS-CoV-2 variant in different countries and different periods is referred to the website:https://covariants.org/per-variant.


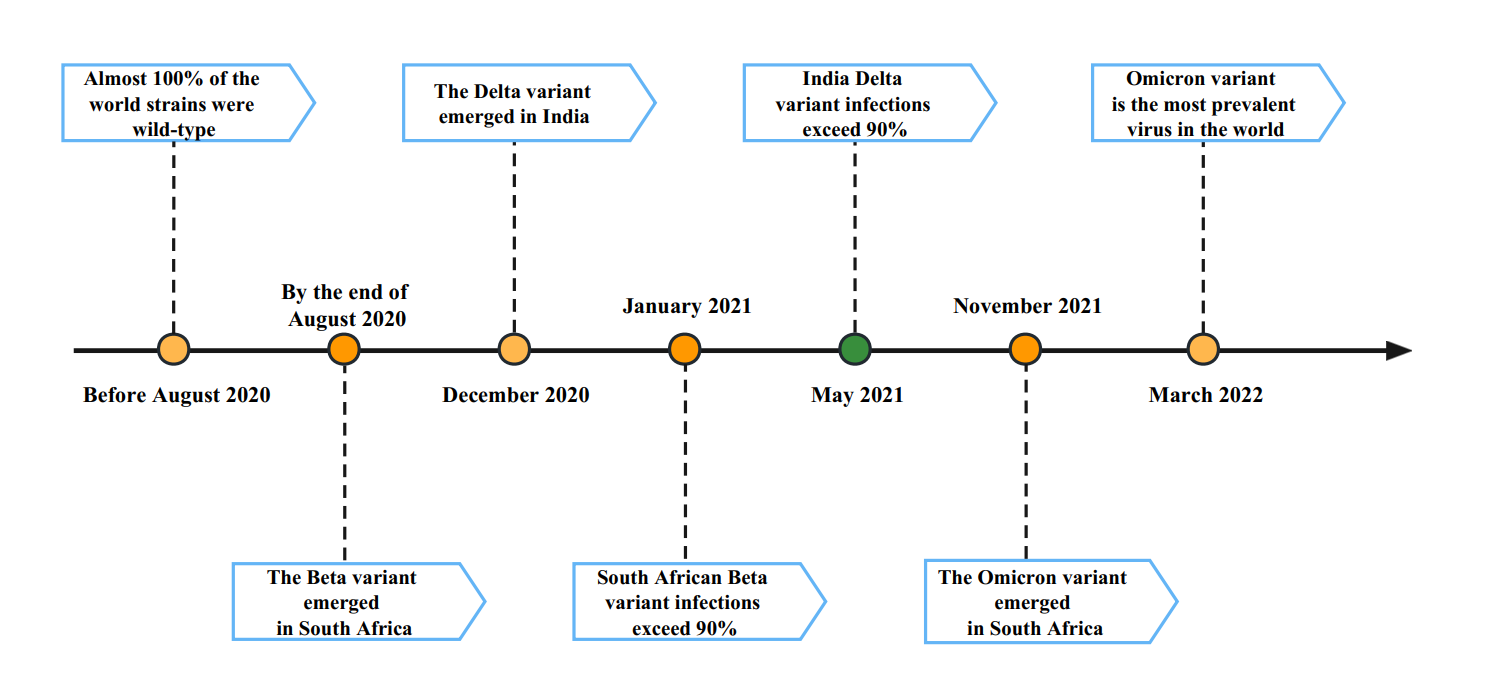


Figure 3-1. Prevalence time of variants.

**Reference**

[1] Bowe B, Xie Y, Al-Aly Z. Acute and postacute sequelae associated with SARS-CoV-2 reinfection. Nat Med 2022;28:2398-2405.

[2] Jeffery-Smith A, Iyanger N, Williams SV, Chow JY, Aiano F, Hoschler K, et al. Antibodies to SARS-CoV-2 protect against re-infection during outbreaks in care homes, September and October 2020. Eurosurveillance 2021;26:2100092.

[3] Vitale J, Mumoli N, Clerici P, De Paschale M, Evangelista I, Cei M, et al. Assessment of SARS-CoV-2 Reinfection 1 Year After Primary Infection in a Population in Lombardy, Italy. JAMA Intern Med 2021;181:1407.

[3] Vitale J, Mumoli N, Clerici P, De Paschale M, Evangelista I, Cei M, et al. Assessment of SARS-CoV-2 Reinfection 1 Year After Primary Infection in a Population in Lombardy, Italy. JAMA Intern Med 2021;181:1407.

[4] Graham MS, Sudre CH, May A, Antonelli M, Murray B, Varsavsky T, et al. Changes in symptomatology, reinfection, and transmissibility associated with the SARS-CoV-2 variant B.1.1.7: an ecological study. Lancet Public Health 2021;6:e335-e345.

[5] Pampa-Espinoza L, Padilla-Rojas C, Silva-Valencia J, Jimenez-Vasquez V, Silva I, Mestanza O, et al. Confirmed Severe Acute Respiratory Syndrome Coronavirus 2 Reinfections After a Second Wave With Predominance of Lambda in Lima and Callao, Peru. Open Forum Infect Dis 2022;9:ofac134.

[6] Malhotra S, Mani K, Lodha R, Bakhshi S, Mathur VP, Gupta P, et al. COVID-19 infection, and reinfection, and vaccine effectiveness against symptomatic infection among health care workers in the setting of omicron variant transmission in New Delhi, India. Lancet Reg Health Southeast Asia 2022;3:100023.

[7] Brouqui P, Colson P, Melenotte C, Houhamdi L, Bedotto M, Devaux C, et al. COVID-19 re-infection. Eur J Clin Invest 2021;51:e13537.

[8] Akpan GE, Bawo L, Amo-Addae M, Kennedy J, Wesseh CS, Whesseh F, et al. COVID-19 reinfection in Liberia: Implication for improving disease surveillance. PLoS One 2022;17:e0265768.

[9] Zare F, Teimouri M, Khosravi A, Rohani-Rasaf M, Chaman R, Hosseinzadeh A, et al. COVID-19 re-infection in Shahroud, Iran: a follow-up study. Epidemiol Infect 2021;149:e159.

[10] Shaheen N, Sambas R, Alenezi M, Alharbi N, Aldibasi O, Bosaeed M. COVID-19 reinfection: A multicenter retrospective study in Saudi Arabia. Ann Thorac Med 2022;17:81-86.

[11] Mensah AA, Lacy J, Stowe J, Seghezzo G, Sachdeva R, Simmons R, et al. Disease severity during SARS-COV-2 reinfection: a nationwide study. J Infect 2022;84:542-550.

[12] Chemaitelly H, Nagelkerke N, Ayoub HH, Coyle P, Tang P, Yassine HM, et al. Duration of immune protection of SARS-CoV-2 natural infection against reinfection. J Travel Med 2022;29:taac109.

[13] Quattrocchi A, Tsioutis C, Demetriou A, Kyprianou T, Athanasiadou M, Silvestros V, et al. Effect of vaccination on SARS-CoV-2 reinfection risk: a case-control study in the Republic of Cyprus. Public Health 2022;204:84-86.

[14] Lewis N, Chambers LC, Chu HT, Fortnam T, De Vito R, Gargano LM, et al. Effectiveness Associated With Vaccination After COVID-19 Recovery in Preventing Reinfection. JAMA Netw Open 2022;5:e2223917.

[15] Hammerman A, Sergienko R, Friger M, Beckenstein T, Peretz A, Netzer D, et al. Effectiveness of the BNT162b2 Vaccine after Recovery from Covid-19. N Engl J Med 2022;386:1221-1229.

[16] Yuguero O, Companys M, Guzmán M, Maciel R, Llobet C, López A, et al. Epidemiological and clinical characteristics of SARS-CoV-2 reinfections in a Spanish region. SAGE Open Med 2022;10:20503121221108556.

[17] Carazo S, Skowronski DM, Brisson M, Sauvageau C, Brousseau N, Gilca R, et al. Estimated Protection of Prior SARS-CoV-2 Infection Against Reinfection With the Omicron Variant Among Messenger RNA-Vaccinated and Nonvaccinated Individuals in Quebec, Canada. AMA Netw Open 2022;5:e2236670.

[18] de Arriba Fernandez A, Bilbao JLA, Frances AE, Mora AC, Perez AG, Barreiros MAD. Evaluation of persistent COVID and SARS-CoV-2 reinfection in a cohort of patients on the island of Gran Canaria, Spain. Semergen 2023;49:101939.

[19] Erbaş İC, Keleş YE, Erdeniz EH, Yılmaz AT, Yeşil E, Çakıcı Ö, et al. Evaluation of possible COVID-19 reinfection in children: A multicenter clinical study. Arch Pediatr 2023;30:187-191.

[20] Pecoraro V, Pirotti T, Trenti T. Evidence of SARS-CoV-2 reinfection: analysis of 35,000 subjects and overview of systematic reviews. Clin Exp Med 2022;1-12.

[21] Bastard J, Taisne B, Figoni J, Mailles A, Durand J, Fayad M, et al. Impact of the Omicron variant on SARS-CoV-2 reinfections in France, March 2021 to February 2022. Eurosurveillance 2022;27:2200247.

[22] Richards J, Rivelli A, Fitzpatrick V, Blair C, Copeland K. Incidence of COVID-19 reinfection among Midwestern healthcare employees. PLoS One 2022;17:e0262164.

[23] Medic S, Anastassopoulou C, Lozanov-Crvenkovic Z, Dragnic N, Petrovic V, Ristic M, et al. Incidence, Risk, and Severity of SARS-CoV-2 Reinfections in Children and Adolescents Between March 2020 and July 2022 in Serbia. JAMA Netw Open 2023;6:e2255779.

[24] Arslan F, Isık Goren B, Baysal B, Vahaboğlu H. Is vaccination necessary for COVID-19 patients? A retrospective cohort study investigating reinfection rates and symptomatology in a tertiary hospital. Expert Rev Vaccines 2022;21:249-252.

[25] Casey SM, Legler A, Hanchate AD, Perkins RB. Likelihood of COVID-19 reinfection in an urban community cohort in Massachusetts. Dialogues Health 2022;1:100057.

[26] Hønge BL, Hindhede L, Kaspersen KA, Harritshøj LH, Mikkelsen S, Holm DK, et al. Long-term detection of SARS-CoV-2 antibodies after infection and risk of re-infection. Int J Infect Dis 2022;116:289-292.

[27] Michlmayr D, Hansen CH, Gubbels SM, Valentiner-Branth P, Bager P, Obel N, et al. Observed protection against SARS-CoV-2 reinfection following a primary infection: A Danish cohort study among unvaccinated using two years of nationwide PCR-test data. LancetT Reg Health-Eur 2022;20:100452.

[28] Dobaño C, Ramírez-Morros A, Alonso S, Vidal-Alaball J, Ruiz-Olalla G, Vidal M, et al. Persistence and baseline determinants of seropositivity and reinfection rates in health care workers up to 12.5 months after COVID-19. BMC Med 2021;19:155.

[29] Rothberg MB, Kim P, Shrestha NK, Kojima L, Tereshchenko LG. Protection Against the Omicron Variant Offered by Previous Severe Acute Respiratory Syndrome Coronavirus 2 Infection: A Retrospective Cohort Study. Clin Infect Dis 2023;76:e142-e147.

[30] Lacy J, Mensah A, Simmons R, Andrews N, Siddiqui MR, Bukasa A, et al. Protective effect of a first SARS-CoV-2 infection from reinfection: a matched retrospective cohort study using PCR testing data in England. Epidemiol Infect 2022;150:e109.

[31] Slezak J, Bruxvoort K, Fischer H, Broder B, Ackerson B, Tartof S. Rate and severity of suspected SARS-Cov-2 reinfection in a cohort of PCR-positive COVID-19 patients. Clin Microbiol Infect 2021;27:1860.e7-1860.e10.

[32] Flacco ME, Acuti Martellucci C, Soldato G, Carota R, Fazii P, Caponetti A, et al. Rate of reinfections after SARS-CoV-2 primary infection in the population of an Italian province: a cohort study. J Public Health 2022;44:e475-e478.

[33] Hurtado IC, Hurtado JS, Valencia SL, Pinzón EM, Guzmán AR, Lesmes MC. Reinfection by SARS CoV2 in Valle Del Cauca, Colombia: A Descriptive Retrospective Study. Inquiry 2022;59:469580221096528.

[34] Prete CA Jr, Buss LF, Buccheri R, Abrahim CMM, Salomon T, Crispim MAE, et al. Reinfection by the SARS-CoV-2 Gamma variant in blood donors in Manaus, Brazil. BMC Infect Dis 2022;22:127.

[35] Arslan Y, Akgul F, Sevim B, Varol ZS, Tekin S. Re-infection in COVID-19: Do we exaggerate our worries? Eur J Clin Invest 2022;52:e13767.

[36] Guedes AR, Oliveira MS, Tavares BM, Luna-Muschi A, Lazari CDS, Montal AC, et al. Reinfection rate in a cohort of healthcare workers over 2 years of the COVID-19 pandemic. Sci Rep 2023;13:712.

[37] Sheehan MM, Reddy AJ, Rothberg MB. Reinfection Rates Among Patients Who Previously Tested Positive for Coronavirus Disease 2019: A Retrospective Cohort Study. Clin Infect Dis 2021;73:1882-1886.

[38] Jeffery-Smith A, Rowland TAJ, Patel M, Whitaker H, Iyanger N, Williams SV, et al. Reinfection with new variants of SARS-CoV-2 after natural infection: a prospective observational cohort in 13 care homes in England. Lancet Healthy Longev 2021;2:e811-e819.

[39] Qureshi AI, Baskett WI, Huang W, Lobanova I, Hasan Naqvi S, Shyu CR. Reinfection With Severe Acute Respiratory Syndrome Coronavirus 2 (SARS-CoV-2) in Patients Undergoing Serial Laboratory Testing. Clin Infect Dis 2022;74:294-300.

[40] Akinbami LJ, Biggerstaff BJ, Chan PA, McGibbon E, Pathela P, Petersen LR. Reinfection With Severe Acute Respiratory Syndrome Coronavirus 2 Among Previously Infected Healthcare Personnel and First Responders. Clin Infect Dis 2022;75:E201-E207.

[41] Alebouyeh M, Aavani P, Abdulrahman NA, Haleem AA, Karimi A, Armin S, et al. Re-positive PCR of SARS-CoV-2 in health care persons during COVID-19 pandemic. Cell Mol Biol Noisy--Gd 2022;67:138-143.

[42] Barzegar M, Manteghinejad A, Bagherieh S, Sindarreh S, Mirmosayyeb O, Javanmard SH, et al. Risk and severity of SARS-CoV-2 reinfection among patients with multiple sclerosis vs. the general population: a population-based study. Mult Scler J 2022;28:518.

[43] Leidi A, Koegler F, Dumont R, Dubos R, Zaballa ME, Piumatti G, et al. Risk of Reinfection After Seroconversion to Severe Acute Respiratory Syndrome Coronavirus 2 (SARS-CoV-2): A Population-based Propensity-score Matched Cohort Study. Clin Infect Dis 2022;74:622-629.

[44] Mensah AA, Campbell H, Stowe J, Seghezzo G, Simmons R, Lacy J, et al. Risk of SARS-CoV-2 reinfections in children: a prospective national surveillance study between January, 2020, and July, 2021, in England. Lancet Child Adolesc Health 2022;6:384-392.

[45] Ozudogru O, Bahce YG, Acer O. SARS CoV-2 reinfection rate is higher in the Omicron variant than in the Alpha and Delta variants. Ir J Med Sci 2022;192:751-756.

[46] Malhotra S, Mani K, Lodha R, Bakhshi S, Mathur VP, Gupta P, et al. SARS-CoV-2 Reinfection Rate and Estimated Effectiveness of the Inactivated Whole Virion Vaccine BBV152 Against Reinfection Among Health Care Workers in New Delhi, India. JAMA Netw Open 2022;5:e2142210.

[47] Wilkins JT, Hirschhorn LR, Gray EL, Wallia A, Carnethon M, Zembower TR, et al. Serologic Status and SARS-CoV-2 Infection over 6 Months of Follow Up in Healthcare Workers in Chicago: A Cohort Study. Infect Control Hosp Epidemiol 2022;43:1207-1215.

[48] Good MK, Czarnik M, Harmon KG, Aukerman D, O'Neal CS, Day C, et al. Severe Acute Respiratory Syndrome Coronavirus 2 (SARS-CoV-2) Infections and Reinfections Among Fully Vaccinated and Unvaccinated University Athletes-15 States, January-November 2021. Clin Infect Dis 2022;75:S236-S242.

[49] Bean DJ, Monroe J, Turcinovic J, Moreau Y, Connor JH, Sagar M. Severe Acute Respiratory Syndrome Coronavirus 2 Reinfection Associates With Unstable Housing and Occurs in the Presence of Antibodies. Clin Infect Dis 2022;75:E208-E215.

[50] Ochoa-Hein E, Leal-Morán PE, Nava-Guzmán KA, Vargas-Fernández AT, Vargas-Fernández JF, Díaz-Rodríguez F, et al. Significant Rise in SARS-CoV-2 Reinfection Rate in Vaccinated Hospital Workers during the Omicron Wave: A Prospective Cohort Study. Rev Invest Clin 2022;74:175-180.

[51] Ando W, Horii T, Jimbo M, Uematsu T, Atsuda K, Hanaki H, et al. Smoking cessation in the elderly as a sign of susceptibility to symptomatic COVID-19 reinfection in the United States. Front Public Health 2022;10:985494.

[52] Lawandi A, Warner S, Sun J, Demirkale CY, Danner RL, Klompas M, et al. Suspected Severe Acute Respiratory Syndrome Coronavirus 2 (SARS-COV-2) Reinfections: Incidence, Predictors, and Healthcare Use Among Patients at 238 US Healthcare Facilities, 1 June 2020 to 28 February 2021. Clin Infect Dis 2022;74:1489-1492.

[53] Gazit S, Shlezinger R, Perez G, Lotan R, Peretz A, Ben-Tov A, et al. The Incidence of SARS-CoV-2 Reinfection in Persons With Naturally Acquired Immunity With and Without Subsequent Receipt of a Single Dose of BNT162b2 Vaccine A Retrospective Cohort Study. Ann Intern Med 2022;175:674-681.

[54] Racine É, Boivin G, Longtin Y, McCormack D, Decaluwe H, Savard P, et al. The REinfection in COVID-19 Estimation of Risk (RECOVER) study: Reinfection and serology dynamics in a cohort of Canadian healthcare workers. Influenza Other Respir Viruses 2022;16:916-925.

[55] Piazza MF, Amicizia D, Marchini F, Astengo M, Grammatico F, Battaglini A, et al. Who Is at Higher Risk of SARS-CoV-2 Reinfection? Results from a Northern Region of Italy. Vaccines 2022;10:1885.
