# Supplementary material for: How does the SARS-CoV-2 reinfection rate change over time? The global evidence from systematic review and meta-analysis
Source: BMC Infect Dis. 2024 Mar 21;24:339. doi: 10.1186/s12879-024-09225-z (PMC10956270; doi:10.1186/s12879-024-09225-z)
Supplement: Supplementary file 4 — Additional file 4: Risk of bias assessments. [file 12879_2024_9225_MOESM4_ESM.docx]

**Additional file 4.** **Risk of bias assessments**

Table 4-1. Cohort study studies table

| Author | Selection | | | | Comparability | Outcome | | | Total number of stars | Quality assessment |
| --- | --- | --- | --- | --- | --- | --- | --- | --- | --- | --- |
|  | Representativeness of the exposed cohort | Selection of the non exposed cohort | Ascertainment of exposure | Demonstration that outcome of interest was not present at start of study | Comparability of cohorts on the basis of the design or analysis | Assessment of outcome | Was follow-up long enough for outcomes to occur | Adequacy of follow up of cohorts |  |  |
| Mark S Graham[4] | ★ | / | / | / | / | / | ★ | ★ | 3 | low |
| Anna Jeffery-Smith[2] | / | / | ★ | / | / | ★ | ★ | ★ | 4 | moderate |
| Luis Pampa-Espinoza[5] | ★ | / | ★ | / | / | ★ | ★ | ★ | 5 | moderate |
| Sumit Malhotra[6] | / | / | / | / | ★★ | / | ★ | ★ | 2 | low |
| Philippe Brouqui[7] | ★ | / | ★ | / | / | ★ | ★ | ★ | 5 | moderate |
| Fariba Zare[9] | ★ | / | ★ | / | / | ★ | ★ | ★ | 5 | moderate |
| Godwin E Akpan[8] | ★ | / | ★ | / | ★ | ★ | ★ | ★ | 6 | moderate |
| Naila A Shaheen[10] | ★ | / | ★ | / | ★ | ★ | ★ | ★ | 6 | moderate |
| Anna A Mensah[11] | ★ | / | ★ | / | ★★ | ★ | ★ | ★ | 7 | high |
| Hiam Chemaitelly[12] | ★ | ★ | ★ | / | ★★ | ★ | ★ | ★ | 8 | high |
| Nickolas Lewis[14] | ★ | / | ★ | / | ★★ | ★ | ★ | ★ | 7 | high |
| Ariel Hammerman[15] | ★ | ★ | ★ | / | ★★ | ★ | ★ | ★ | 8 | high |
| Oriol Yuguero[16] | ★ | / | ★ | / | / | ★ | ★ | / | 4 | moderate |
| A. de Arriba Fernández[18] | ★ | / | ★ | / | ★★ | ★ | ★ | ★ | 7 | high |
| Lara J Akinbami[40] | ★ | / | ★ | / | ★★ | ★ | ★ | ★ | 7 | high |
| Yusuf Arslan[35] | ★ | / | ★ | / | / | ★ | ★ | ★ | 5 | moderate |
| Carlota Dobaño[28] | / | ★ | ★ | ★ | / | ★ | ★ | ★ | 6 | moderate |
| M E Flacco[32] | ★ | ★ | ★ | ★ | / | ★ | ★ | ★ | 7 | high |
| Ana Rubia Guedes[36] | / | ★ | ★ | ★ | / | ★ | ★ | ★ | 6 | moderate |
| Anna Jeffery-Smith[38] | / | ★ | ★ | ★ | ★★ | ★ | ★ | ★ | 8 | high |
| Joanne Lacy[30] | ★ | ★ | ★ | ★ | ★★ | ★ | ★ | ★ | 9 | high |
| Daniela Michlmayr[27] | ★ | ★ | ★ | ★ | / | ★ | ★ | / | 6 | moderate |
| Carlos A Prete Jr[34] | ★ | ★ | ★ | ★ | / | ★ | ★ | ★ | 7 | high |
| Adnan I Qureshi[39] | ★ | ★ | ★ | ★ | / | ★ | ★ | / | 6 | moderate |
| Étienne Racine[54] | / | ★ | ★ | ★ | / | ★ | ★ | ★ | 6 | moderate |
| Michael B Rothberg[29] | ★ | ★ | ★ | ★ | ★★ | ★ | ★ | / | 8 | high |
| megan M[37] | ★ | ★ | ★ | ★ | ★ | ★ | ★ | ★ | 8 | high |
| Laith J. Abu-Raddad[62] | / | ★ | ★ | ★ | ★★ | ★ | / | ★ | 7 | high |
| Sumit Malhotra[46] | / | ★ | / | ★ | ★★ | ★ | ★ | ★ | 7 | high |
| Antonio Leidi[43] | ★ | ★ | ★ | ★ | ★★ | ★ | ★ | ★ | 9 | high |
| Eric Ochoa-Hein[50] | / | ★ | ★ | ★ | ★★ | ★ | ★ | ★ | 8 | high |
| Alexander Lawandi[52] | ★ | ★ | ★ | ★ | ★★ | ★ | ★ | ★ | 9 | high |
| Ferhat Arslan[24] | ★ | ★ | ★ | ★ | / | ★ | ★ | ★ | 7 | high |
| Sharon M. Casey[25] | ★ | ★ | ★ | ★ | ★★ | ★ | ★ | ★ | 9 | high |
| İrem Ceren Erbaş[10] | ★ | ★ | ★ | ★ | / | ★ | ★ | ★ | 7 | high |
| S. Gazit[53] | ★ | ★ | ★ | ★ | / | ★ | ★ | ★ | 7 | high |
| B. L. Hønge[26] | ★ | ★ | ★ | ★ | ★★ | ★ | ★ | ★ | 9 | high |
| S. Medic[23] | ★ | ★ | ★ | ★ | / | ★ | ★ | ★ | 7 | high |
| J. Richards[22] | ★ | ★ | ★ | ★ | / | ★ | ★ | ★ | 7 | high |

Table 4-2. Case-control study assessment table

| Author | Selection | | | | Comparability | Ecpose | | | Total number of stars | Quality assessment |
| --- | --- | --- | --- | --- | --- | --- | --- | --- | --- | --- |
|  | Is the case definition adequate | Representativeness of the cases | Selection of Controls | Definition of Controls | Comparability of cases and controls on the basis of the design or analysis | Ascertainment of exposure | Same method of ascertainment for cases and controls | Non-Response rate |  |  |
| Benjamin Bowe[1] | ★ | ★ | ★ | ★ | / | ★ | ★ | / | 6 | moderate |
| Josè Vitale[3] | ★ | ★ | / | ★ | ★★ | ★ | ★ | / | 7 | high |
| Sara Carazo[17] | ★ | ★ | ★ | ★ | ★★ | ★ | ★ | ★ | 9 | high |
| Anna A Mensah[13] | ★ | ★ | ★ | ★ | / | ★ | ★ | ★ | 7 | high |
| David J Bean[49] | ★ | / | / | ★ | / | ★ | ★ | ★ | 5 | moderate |
| Maria Francesca Piazza[55] | ★ | ★ | ★ | ★ | / | ★ | ★ | ★ | 7 | high |
| Mahdi Barzegar[42] | ★ | ★ | ★ | ★ | ★★ | ★ | ★ | ★ | 9 | high |

Table 4-3. Cross-sectional study assessment table

| Author | Were the criteria for inclusion in the sample clearly defined? | Were the study subjects and the setting described in detail? | Was the exposure measured in a valid and reliable way? | Were objective, standard criteria used for measurement of the condition? | Were confounding factors identified? | Were strategies to deal with confounding factors stated? | Were the outcomes measured in a valid and reliable way? | Was appropriate statistical analysis used? | The number of “Yes” | quality assessment |
| --- | --- | --- | --- | --- | --- | --- | --- | --- | --- | --- |
| Isabel Cristina Hurtado[33] | Yes | Yes | Yes | Yes | No | No | Yes | Yes | 6 | Inclusion |
| Osman Özüdoğru[45] | Yes | No | Yes | Yes | No | No | Yes | Yes | 4 | Exclusion |
| Mary K Good[48] | Yes | Yes | Yes | Yes | Yes | Yes | Yes | Yes | 8 | Inclusion |
| Wataru Ando[51] | Yes | Yes | Yes | Yes | Yes | Yes | Yes | Yes | 8 | Inclusion |
| Jonathan Bastard[21] | Yes | Yes | Yes | Yes | Unclear | No | Yes | Unclear | 5 | Inclusion |
| Anna A Mensah[44] | Yes | Yes | Yes | Yes | Yes | Yes | Yes | Yes | 8 | Inclusion |
| Masoud Alebouyeh[41] | Yes | No | Yes | Yes | No | No | Yes | No | 4 | Exclusion |
| Valentina Pecoraro[20] | Yes | Yes | Yes | Yes | Yes | No | Yes | No | 6 | Inclusion |
