# Supplementary material for: How does the SARS-CoV-2 reinfection rate change over time? The global evidence from systematic review and meta-analysis
Source: BMC Infect Dis. 2024 Mar 21;24:339. doi: 10.1186/s12879-024-09225-z (PMC10956270; doi:10.1186/s12879-024-09225-z)
Supplement: Supplementary file 5 — Additional file 5: Distribute transformation results. [file 12879_2024_9225_MOESM5_ESM.docx]

**Additional file 5.** **Distribute transformation results**

Table 5-1 Normal test for different rate transformations (All data)

Results of Shapiro-Wilk normality test

| Transformation type | W value | *P* value |
| --- | --- | --- |
| raw data | 0.69379 | 2.263*10^-7^ |
| log | 0.97847 | 0.6934 |
| logit | 0.97965 | 0.7335 |
| arcsin | 0.88330 | 0.0012 |
| darcsin | 0.88365 | 0.0013 |
