# Supplementary material for: How does the SARS-CoV-2 reinfection rate change over time? The global evidence from systematic review and meta-analysis
Source: BMC Infect Dis. 2024 Mar 21;24:339. doi: 10.1186/s12879-024-09225-z (PMC10956270; doi:10.1186/s12879-024-09225-z)
Supplement: Supplementary file 6 — Additional file 6: Meta-analysis results. [file 12879_2024_9225_MOESM6_ESM.docx]

**Additional file 6.** **Meta-analysis results**


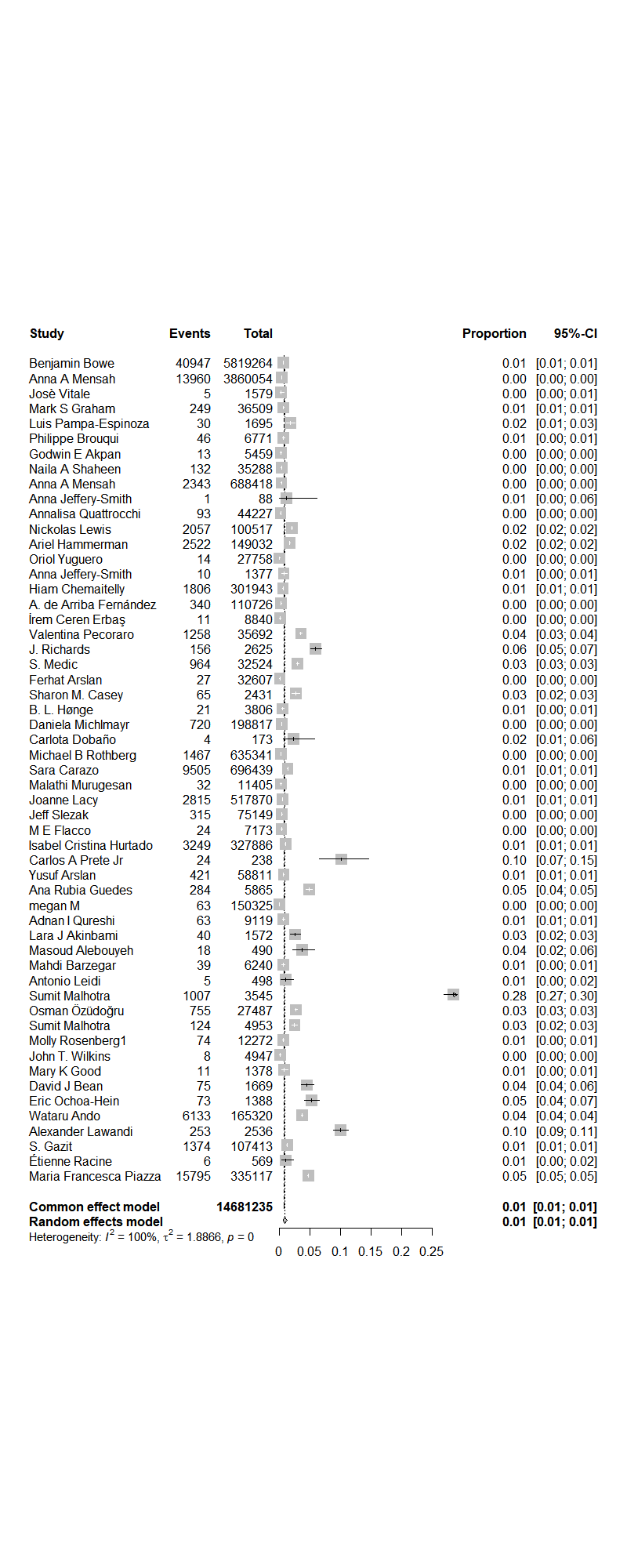


Figure 6-1. General forest map.


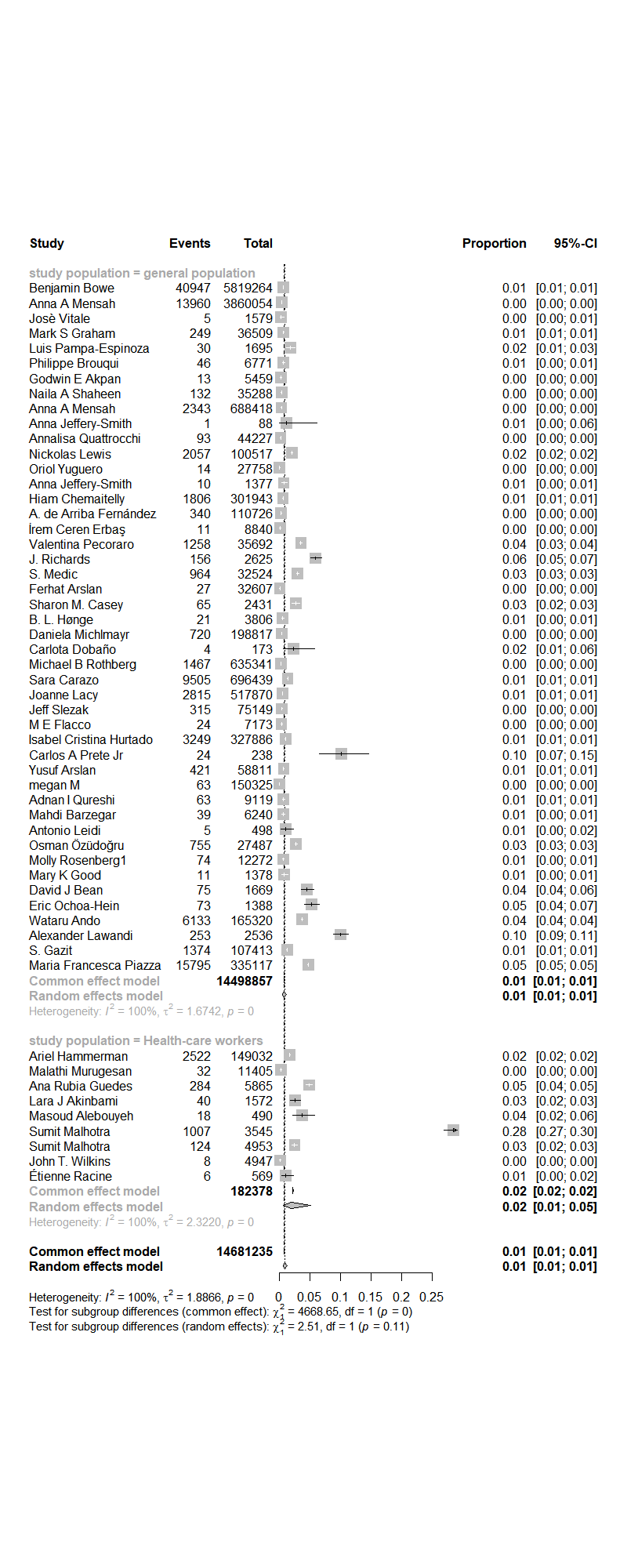


Figure 6-2. Forest plot for subgroup analysis of the study population.


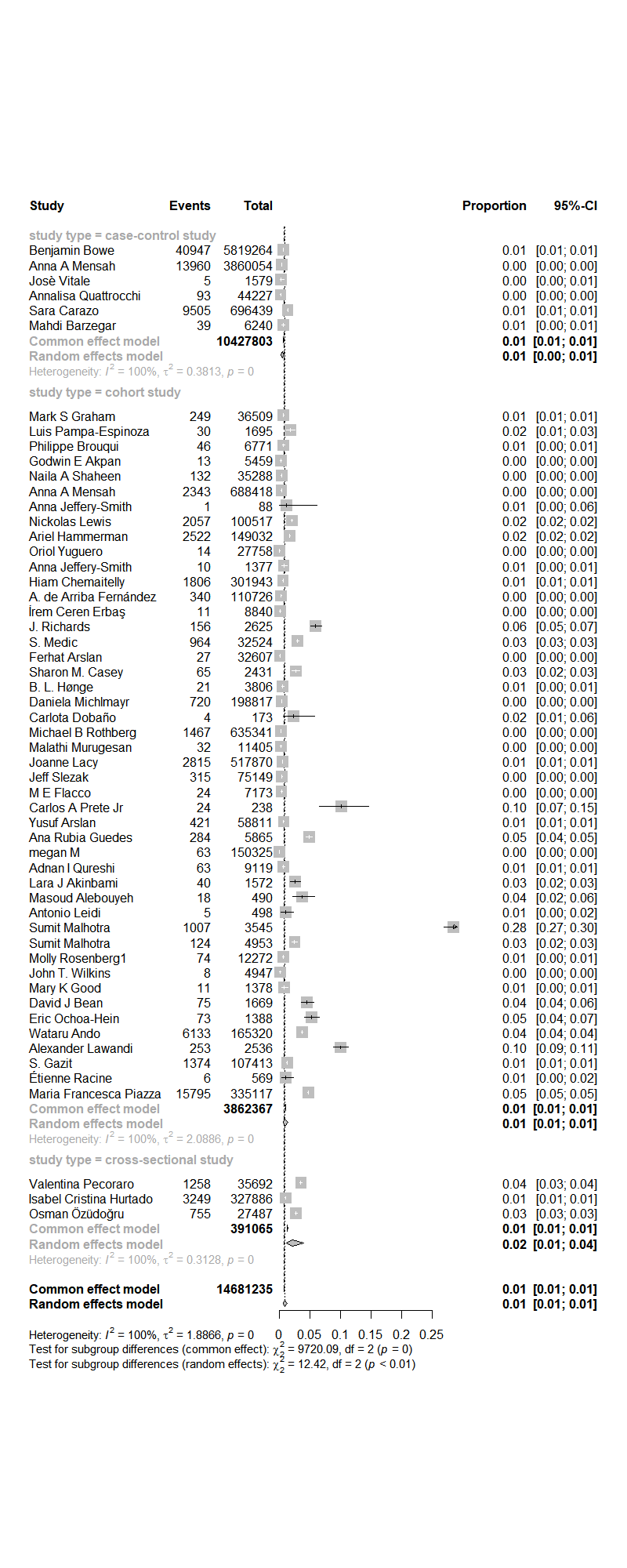


Figure 6-3. Forest plot for subgroup analysis of study type.


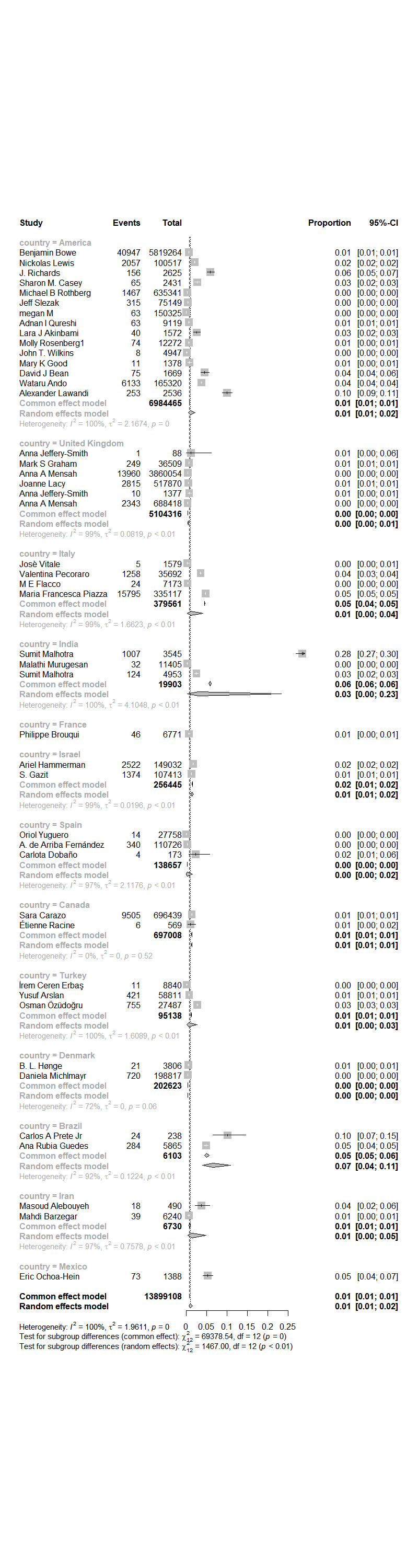


Figure 6-4. Forest map for national subgroup analysis.


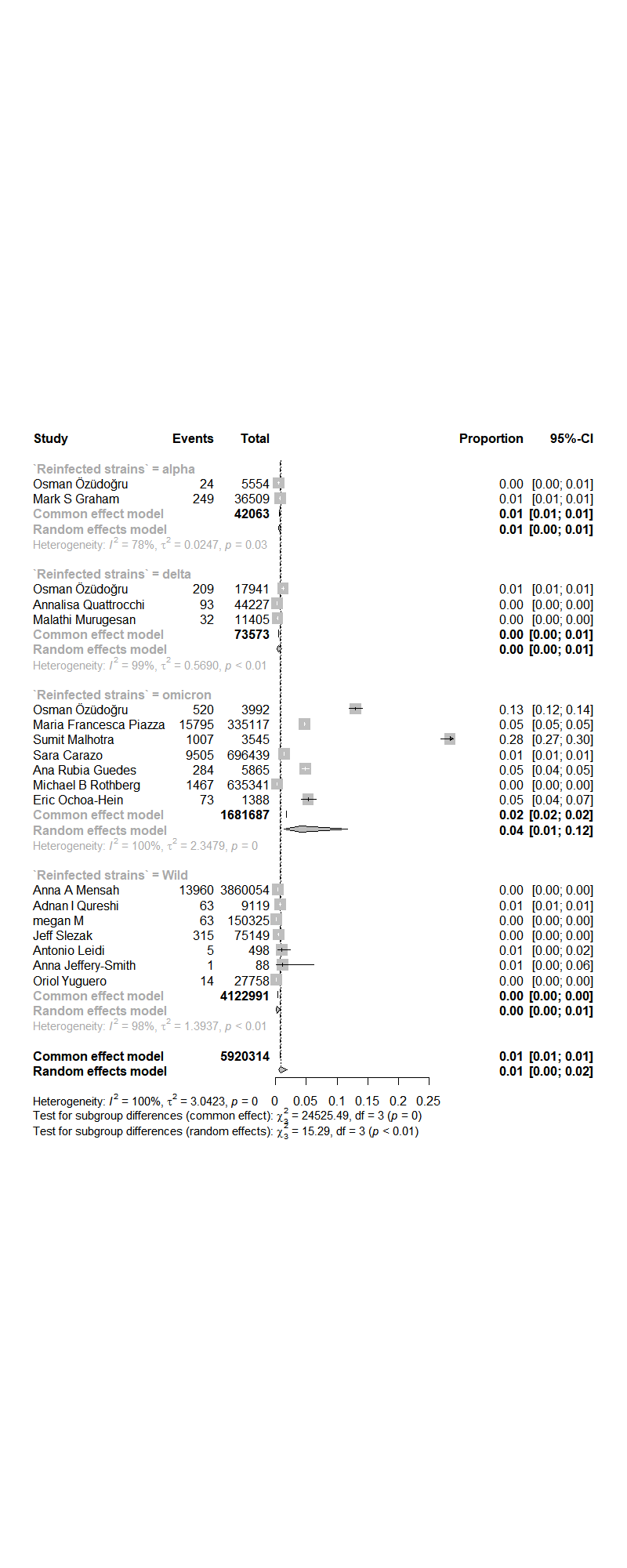


Figure 6-5. Forest plot for subgroup analysis of reinfection strain.

.


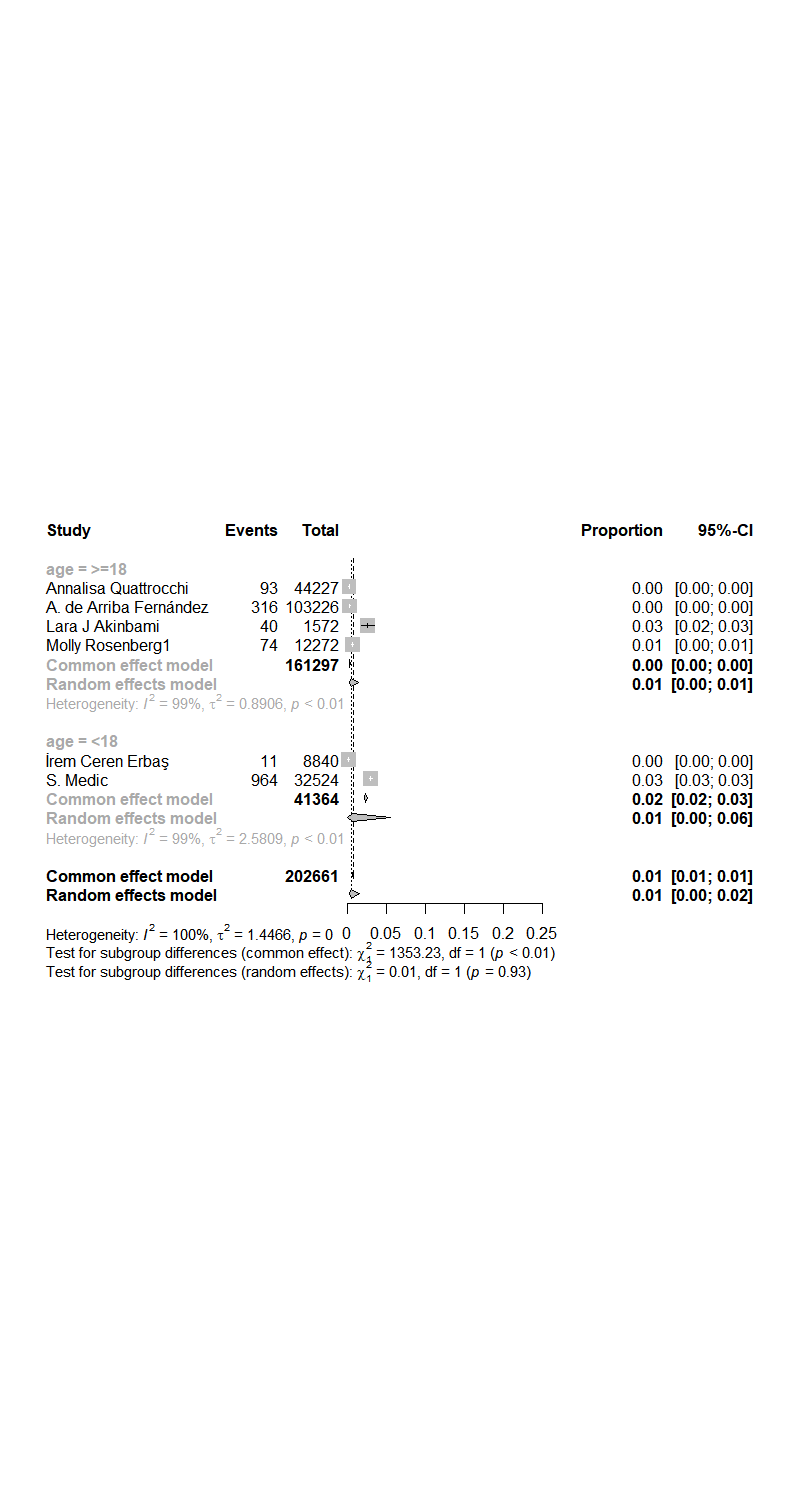


Figure 6-6. Forest plot for subgroup analysis of age.
