# Supplementary material for: How does the SARS-CoV-2 reinfection rate change over time? The global evidence from systematic review and meta-analysis
Source: BMC Infect Dis. 2024 Mar 21;24:339. doi: 10.1186/s12879-024-09225-z (PMC10956270; doi:10.1186/s12879-024-09225-z)
Supplement: Supplementary file 7 — Additional file 7: The AIC of model. [file 12879_2024_9225_MOESM7_ESM.docx]

**Additional file 7.** **The AIC of model**

Table 7-1. AIC of meta-regression of time-varying reinfection rate.

| Model type | AIC |
| --- | --- |
| ~ bs(day, df = 3) + variant | 388.669 |
| ~ bs(day, df = 4) + variant | 382.6962 |
| ~ bs(day, df = 5) + variant | 379.8534 |
| ~ bs(day, df = 6) + variant | 379.0613 |
| ~ bs(day, df = 7) + variant | 377.4777 |
| ~ bs(day, df = 8) + variant | 370.1893 |
| ~ bs(day, df = 9) + variant | 372.55 |
| ~ bs(day, df = 8) + variant + country + studytype | misconvergence |
