# Supplementary material for: How does the SARS-CoV-2 reinfection rate change over time? The global evidence from systematic review and meta-analysis
Source: BMC Infect Dis. 2024 Mar 21;24:339. doi: 10.1186/s12879-024-09225-z (PMC10956270; doi:10.1186/s12879-024-09225-z)
Supplement: Supplementary file 8 — Additional file 8: Sensitivity analysis results. [file 12879_2024_9225_MOESM8_ESM.docx]

**Additional file 8.** **Sensitivity analysis results**


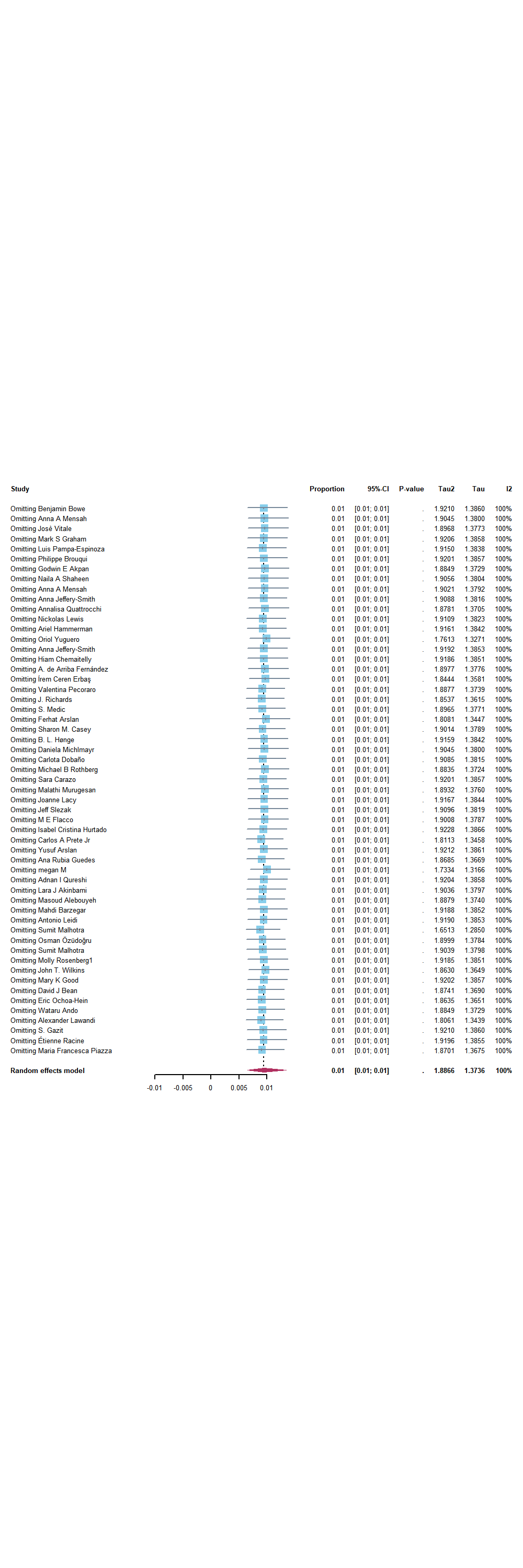


Figure 8-1. Forest plot with sequentially eliminated documents.


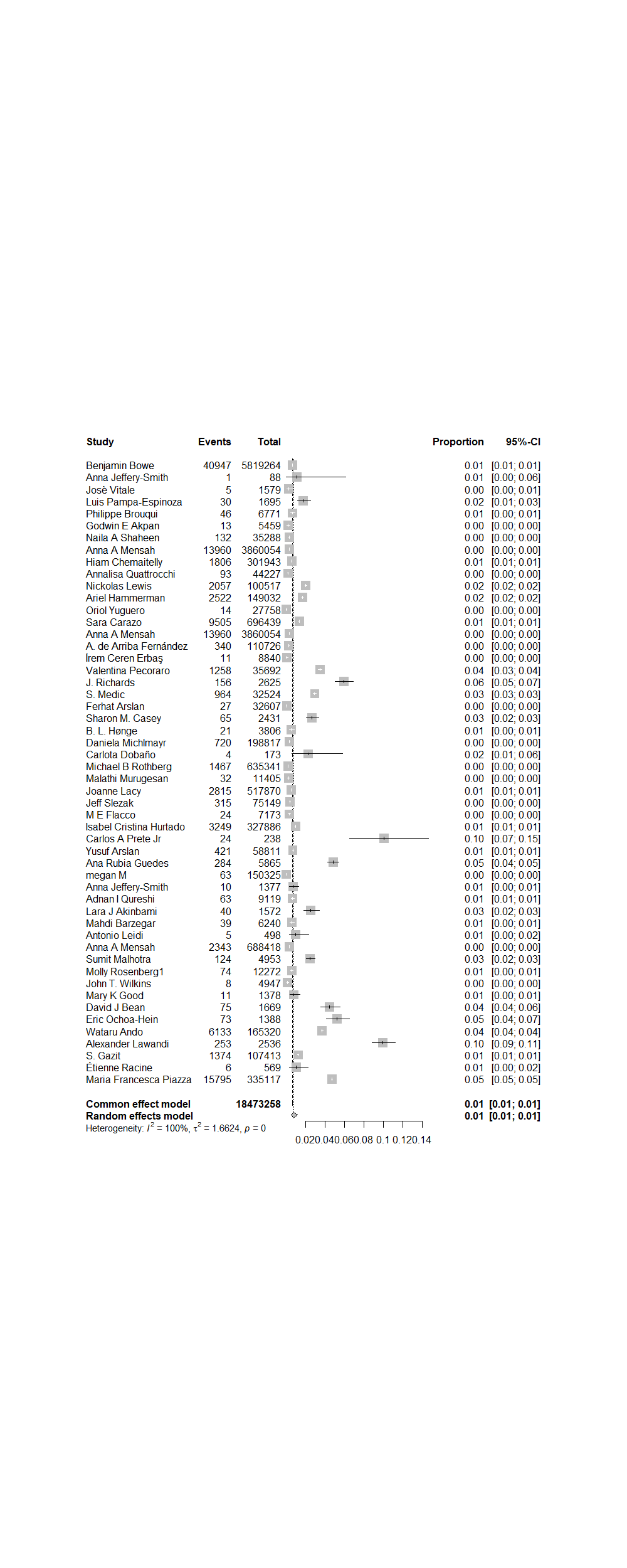


Figure 8-2. Forest maps with high bias literature excluded.
