# Supplementary material for: How does the SARS-CoV-2 reinfection rate change over time? The global evidence from systematic review and meta-analysis
Source: BMC Infect Dis. 2024 Mar 21;24:339. doi: 10.1186/s12879-024-09225-z (PMC10956270; doi:10.1186/s12879-024-09225-z)
Supplement: Supplementary file 9 — Additional file 9: Spline regression of reinfection rate. [file 12879_2024_9225_MOESM9_ESM.docx]

**Additional file 9.** **Spline regression of reinfection rate**


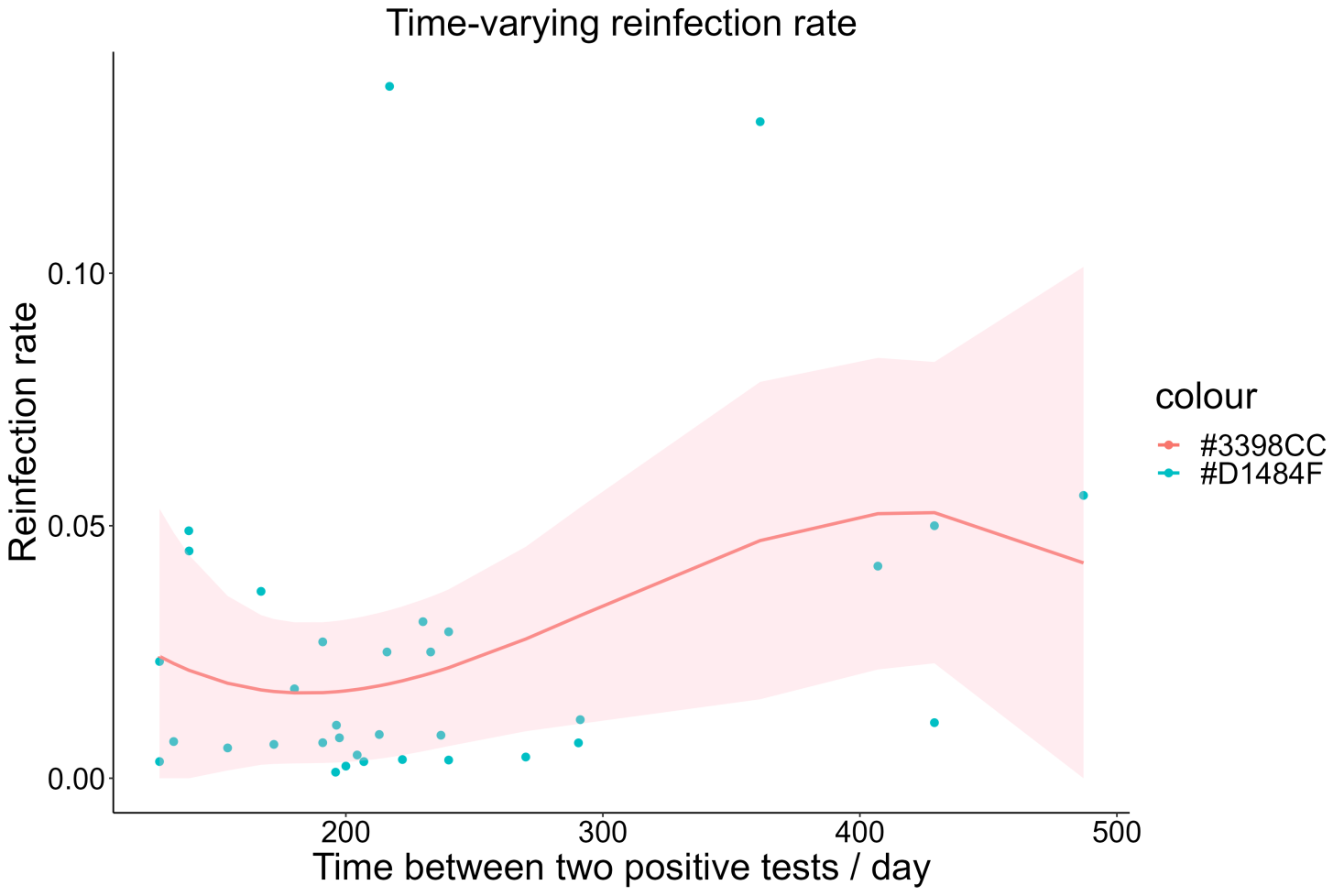


Figure 9-1. Natural spline regression of time-varying reinfection rates (blue dots represent the true values, and shaded part is 95% *CI*).


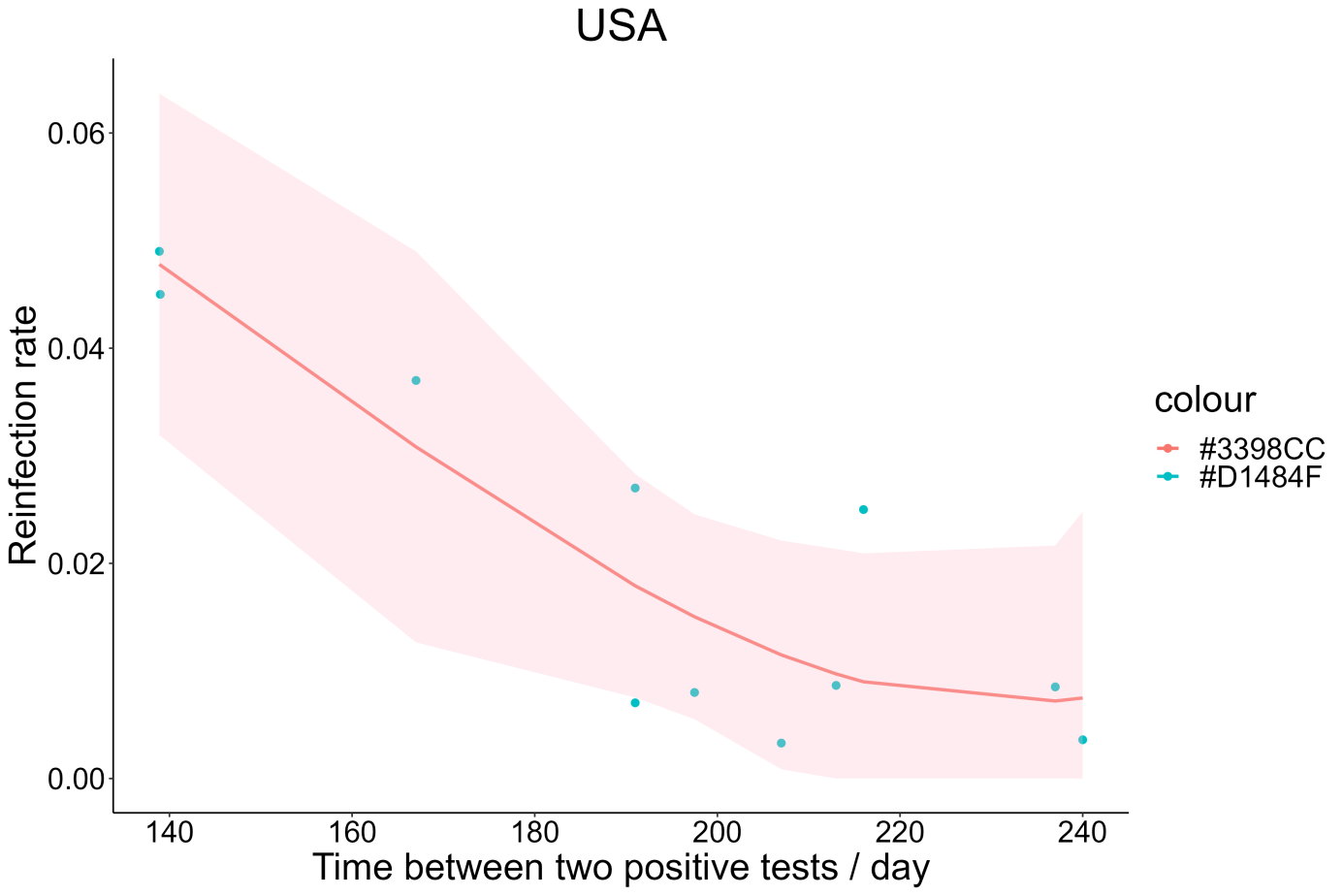


Figure 9-2. Natural spline regression of time-varying reinfection rates in America (blue dots represent the true values, and shaded part is 95% *CI*).


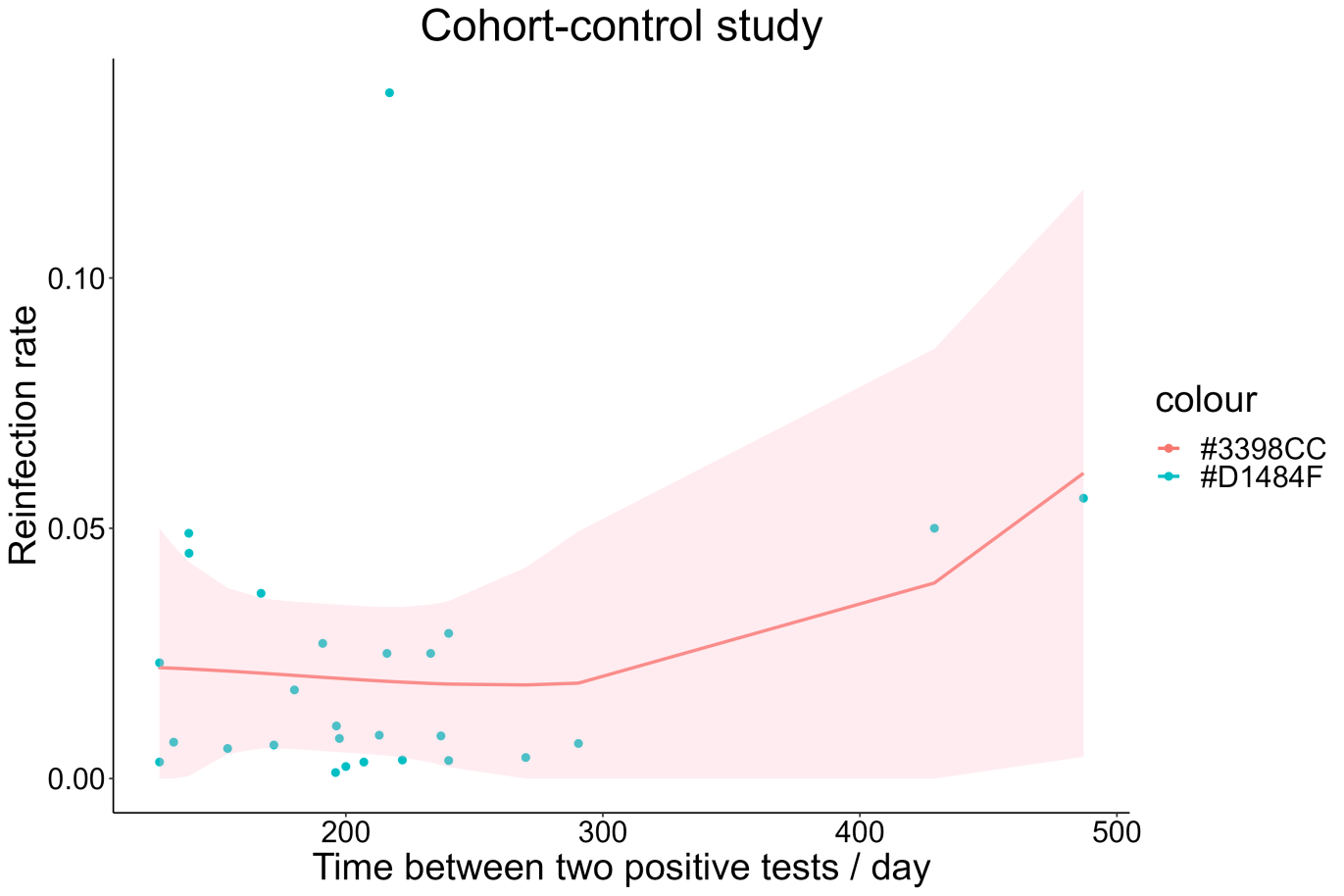


Figure 9-3. Natural spline regression for cohort study (blue dots represent the true values, and shaded part is 95% *CI*).


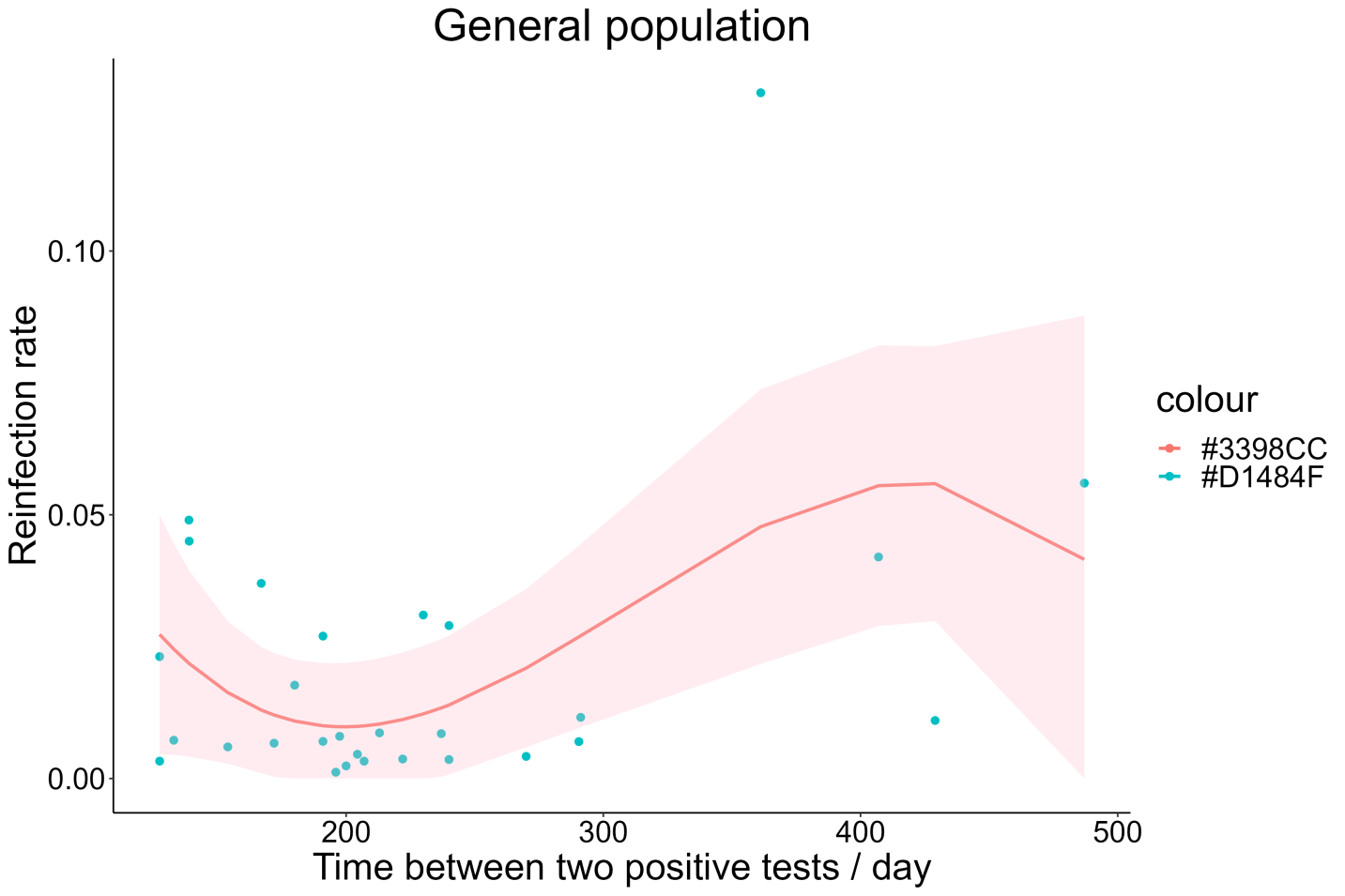


Figure 9-4. Natural spline regression of time-varying reinfection rates in the general population (blue dots represent the true values, and shaded part is 95% *CI*).
